# Synergistic lethality of combination treatment with Trop2-directed antibody-drug conjugate sacituzumab govitecan and TRAIL agonists in triple negative breast cancer

**DOI:** 10.1186/s13058-026-02318-4

**Published:** 2026-08-14

**Authors:** Yonit A. Addissie, Yoshimi E. Greer, Jayakumar Nair, Stanley Lipkowitz

**Affiliations:** Women’s Malignancies Branch, Center for Cancer Research, National Cancer Institute, Bethesda, MD USA

**Keywords:** Triple negative breast cancer, Trop2, Sacituzumab govitecan, IMMU-132, Irinotecan, SN-38, TRAIL, Apo2L, MEDI3039

## Abstract

**Background:**

Sacituzumab govitecan (SG) is an antibody drug conjugate targeting trophoblast cell surface antigen 2 (Trop2) that is approved for treatment of patients with metastatic triple negative breast cancer (TNBC). Tumor necrosis factor-related apoptosis inducing ligand (TRAIL) agonists are antitumor agents that interact with death receptors on the cell surface to induce apoptosis in cancer cells, often sparing normal cells. In this study, we investigated whether combination treatment with SG and TRAIL agonists inhibit TNBC cell growth.

**Methods:**

In vitro, 10 human TNBC cell lines and 4 non-modified, low passage patient derived TNBC cells were treated with SG and the TRAIL agonist Apo2L. Cell death was assessed via a propidium iodide-based assay while cell proliferation was measured using an ATP cell viability assay. The mode of cell death elicited by combination treatment was investigated by using inhibitors of apoptosis, necroptosis and ferroptosis. The drug effect on cell cycle was analyzed with flow cytometry. In vivo, NCr athymic nude (nu/nu) female mice bearing HCC1806 TNBC xenografts were treated with SG and TRAIL agonists after which the effect of treatment on tumor growth and survival was evaluated.

**Results:**

SG and TRAIL acted synergistically in all 10 TNBC cell lines as well as all 4 patient derived TNBC cells tested. Synergistic cell death and growth inhibition was observed in both Trop2 high-expressing and low-expressing cells, with data suggesting that the linker deconjugation and bystander effects of SG contribute to the efficacy in Trop2 low-expressing cells. Caspase-3/7 assays as well as cell death assays using cell death inhibitors demonstrated that the lethality of dual treatment is primarily mediated by apoptosis. Additionally, SG alone and in combination with Apo2L induced cycle arrest at the G2/M phase. Finally, xenograft models using HCC1806 bearing mice showed that dual treatment with SG and TRAIL agonists significantly inhibited tumor growth and caused a borderline significant benefit on survival with no toxicities noted for both drug treatments.

**Conclusions:**

Combination treatment with SG and TRAIL agonists induces synergistic lethality in TNBC cells in vitro and inhibits tumor growth in vivo, suggesting a potential clinical benefit of the combination therapy in TNBC.

**Supplementary Information:**

The online version contains supplementary material available at 10.1186/s13058-026-02318-4.

## Introduction

Breast cancer is the most common malignancy in women after skin cancer, with approximately 2.3 million new cases diagnosed in 2022 and 670,000 deaths reported globally [1]. Triple negative breast cancer (TNBC) accounts for 10–20% of all breast cancers and is characterized by < 1% cellular expression of estrogen receptor and progesterone receptor as well as lack of HER2 amplification [2, 3]. Of the subtypes of breast cancer, TNBC is considered the most aggressive with higher risks of recurrence, metastasis and mortality [4–6]. Patients with TNBC have fewer targeted options compared to other subtypes of breast cancer. One molecular target being studied for TNBC therapy is human trophoblast cell surface antigen 2 (Trop2), which is a monomeric transmembrane calcium signal transducer that is highly expressed in a variety of tumors including prostate, pancreatic and breast cancers [7–9]. Increased Trop2 expression is found in as many as 93% of TNBC, making it a particularly attractive target as monotherapy or as part of combination therapy [10].

Trop2-targeting antibody drug conjugates (ADCs) have been investigated for the treatment of various cancers, with the ADC sacituzumab govitecan (SG) being approved by the FDA for the treatment of metastatic TNBC [11]. SG conjugates a Trop2 binding antibody to the topoisomerase I inhibitor SN-38 (the active metabolite of irinotecan), which induces double stranded breaks in DNA. After binding to surface Trop2, SG gets internalized and allows for targeted delivery of SN-38 to tumor cells [12]. In addition, the membrane permeable SN-38 can elicit antitumor effects against nearby (bystander) cells through extracellular cleavage of the hydrolysable linker before internalization as well as release of the drug after internalization, further enhancing its potency [12–18]. Compared to irinotecan, the antibody drug conjugate allows targeted and enhanced delivery and efficacy of the SN-38 drug to Trop2 expressing tumors in in vivo models [14, 19].

In the ASCENT trial, treatment of TNBC patients with SG significantly improved the progression free survival, overall response rate, and overall survival compared to chemotherapy [20, 21]. While SG monotherapy benefits patients with metastatic TNBC, combination studies of SG are warranted to improve on these results. Efforts to enhance the efficacy of SG have included combination treatment of SG with other anti-tumor agents [22, 23]. In a previous study, a synergistic inhibitory effect of dual treatment with SG and PARP inhibitors was observed with significant antitumor effects and survival benefit found with dual treatment compared to monotherapy in animal models of TNBC [22]. The current study aims to assess the cytotoxic effects of dual treatment with Trop2-directed ADC SG and tumor necrosis factor-related apoptosis-inducing ligand (TRAIL) agonists in TNBC.

TRAIL is a member of the tumor necrosis factor (TNF) gene superfamily that interacts with death receptors to induce apoptosis in various tumor cells including TNBC, often sparing normal cells [24]. We previously demonstrated that mesenchymal TNBC breast cancer cells are very sensitive to TRAIL-induced apoptosis while other subtypes of breast cancer are relatively resistant [25–27]. We have also reported that resistant cells could be sensitized to TRAIL-mediated apoptosis by sub-therapeutic doses of chemotherapeutic drugs and targeted agents such as trastuzumab or EGFR inhibitors [28–32]. Moreover, an enhanced killing effect of TRAIL was observed when combined with topoisomerase I inhibitors in vitro and in vivo in TNBC breast cancer models [31]. In preclinical and clinical trials, TRAIL agonists have been well tolerated and importantly do not induce toxicities typically associated with topoisomerase inhibitors (e.g., hematologic and gastrointestinal toxicities), making them promising agents to be combined with SG [33, 34].

The current study aimed to assess the cytotoxic effects of dual treatment with Trop2-directed ADC SG and TRAIL agonists in TNBC. In vitro studies utilizing 10 TNBC cell lines, 4 primary patient derived TNBC cells confer a synergistic lethality against TNBC and in vivo combination experiments demonstrated anti-tumor activity of the dual treatment with SG and TRAIL. Our results also show that the primary mechanism of cell death for TRAIL and SG combination is apoptosis. Additionally, SG alone and in combination with TRAIL induces a G2/M cell cycle arrest. Overall, our data suggests that dual treatment with SG and TRAIL generates a synergistic effect in killing TNBC cells, highlighting a potential clinical benefit for this combination.

## Materials and methods

### Cell lines and materials

Human TNBC cell lines MDA-MB-231, MDA-MB-468, Hs578T, BT-549, HCC1806, HCC70, MDA-MB-436, and HCC1143 were acquired from the American Type Culture Collection (ATCC; Manassas, VA). BT-20 cell line was obtained from Reinhard Ebner (Avalon Pharmaceuticals, Germantown, MD). SUM-159 was a gift from Dr. Lee Graves, University of North Carolina. ER+/HER2- T47D cells were acquired from ATCC while MCF7 cells were a gift from Dr. Senthil K. Muthuswamy, National Cancer Institute, National Institutes of Health. Cells were maintained in RPMI 1640 base media (except for HS578T - DMEM base media), 10% fetal bovine serum (FBS) and 1% penicillin/streptomycin, and grown at 37 °C, 5% CO_2_. SUM-159 cells were maintained in DMEM/F12 supplemented with 5% FBS, 5 µg/mL insulin, 1 µg/mL hydrocortisone and 1% penicillin/streptomycin, Cell lines were authenticated using Promega GenePrint 10 system or PowerPlex 16 HS System at Laragen Inc. (Culver City, CA). Routine mycoplasma tests were conducted using a mycoplasma PCR detection kit (cat# MP0035, Sigma-Aldrich).

In additional to long-term immortalized human TNBC cell lines above, non-immortalized, low passage patient derived TNBC cells (PDCs) were also used. PDCs with clinically annotated molecular information were obtained from the National Cancer Institute’s Patient Derived Models Repository (PDMR). Four TNBC PDCs were selected: model# 879694-015-R-J1-PDC, 428986-081-R-J1-PDC, K33807-207-R-J1-PDC, 885512-296-R-J2-PDC [35]. Hereafter, the PDCs are referred to as R015, R081, R207 and R296, respectively. PDCs were initially cultured, sub-cultured and cryopreserved using the reagents and materials suggested as well as instructions provided by PDMR [35].

### Reagents

For in vitro studies, SG (brand name Trodelvy) was purchased from the National Institutes of Health Division of Veterinary Resources (DVR) pharmacy (NDC# 55135-132-01) as well as Selleck Chemicals (cat# D4002). Apo2L (commercially available recombinant TRAIL) was purchased from PeproTech (cat# 310-04, Cranbury, NJ). MEDI3039 was provided by MedImmune (Gaithersburg, MD) [25]. SG from DVR, Apo2L and MEDI3039 were used for in vivo experiments. Pan-caspase inhibitor Z-VAD-FMK (cat# S7023), necrostatin (cat# S8037), SN-38 (cat# S4908), carboplatin (cat# S1215) were obtained from Selleck Chemicals, and ferrostatin (cat# HY-100579) was from MedChemExpress. Propidium iodide was purchased from Millipore-Sigma (cat# P4864). RNAse A was from ThermoFisher Scientific (cat# 12091021).

### Trop2, DR4 and DR5 surface expression in TNBC cells

Surface expression of Trop2 was assessed by flow cytometry. Briefly, 5 × 10^5^ cells were harvested and washed with cold sterile Ca^2+^/Mg^2+^-free phosphate-buffered saline (PBS) and incubated with PE-conjugated anti-Trop2 antibody (Invitrogen, cat# 12-6024-42) in FACS buffer (PBS with 10% FBS) as instructed by the manufacturer. Cells were incubated at 4° C for 30 min then washed twice with FACS buffer before being analyzed using a BD LSRFortessa™ (BD Biosciences) flow cytometer. QuantiBRITE PE beads (cat# 340495, BD Biosciences) were run on the same settings to estimate the number of Trop2 molecules per cell. Data was analyzed using FlowJo^®^ software (BD Biosciences).

To measure changes in DR4 and DR5 surface expression with SG and Apo2L treatment, TNBC cells were stained with DR4 (R&D Systems, cat# FAB347A), DR5 (FAB6311G) or isotype controls (cat# IC0041G, IC002A) and analyzed as described above.

### Cell death and cell viability assays

Cell death was assessed using propidium iodide (PI) as previously described [36]. Briefly, 6,000 cells per well were plated in 96-well plates and treated the following day with SG, Apo2L or combination conditions in 10% FBS media supplemented with PI (1:3000 dilution). Brightfield cell number as well as dead cells (PI positive cells) were quantified using a BioTek Cytation 1 plate reader. Plates were read at time 0 and re-read after 24–48 h incubation to quantify cell death percentage ((PI positive cells/total brightfield cell number)*100). Cell viability was measured using CellTiter-Glo 2.0 (cat# G9242) as directed by Promega. Briefly, 2,500 cells per well were plated in a 96-well plates and treated on the following day. Plates were incubated for 24–48 h, then CellTiter-Glo 2.0 assay reagent was added to each well, followed by plate shaking for 2 min and incubation for 10 min before reading using a SpectraMax^®^ iD3 microplate reader. A minimum of three independent experiments were carried out per cell line. Drug interaction was determined using the online SynergyFinder tool where synergy scores are calculated using four computational models to determine if drug combination is synergistic (score > 10), additive (score − 10 to 10) or antagonistic (score < -10) [37]. Drug doses were chosen and optimized after conducting IC50-finding assays using CellTiter-Glo 2.0 and identifying the different sensitives of the cell lines to SG and TRAIL/Apo2L. Dosages were chosen so that single drug treatment did not have a potent enough effect to mask the effects of combination treatment.

### Caspase 3/7 activity assay

Activation of caspase 3/7 was assessed as previously described with Promega’s Caspase-Glo 3/7 assay (cat# G8093) as directed by Promega [38]. Luminescence was read using a SpectraMax^®^ iD3 microplate reader.

### Annexin V apoptosis analysis

Flow cytometry-based annexin V assay was performed to assess apoptosis induction. Cells were plated in 6-well plates (5 × 10^5^ cells per well) and treated with either SG, Apo2L or both drugs. Cells were harvested 24 h later and washed twice with cold PBS before being stained with 7-AAD and annexin V using the eBioscience™ Annexin V Apoptosis Detection Kit as directed (cat# 88-8006-74, Thermo Fisher Scientific). Cells were analyzed on a BD LSRFortessa™ flow cytometer and the data was analyzed using FlowJo^®^ software. Early apoptotic cells were identified by positive annexin V staining and negative 7-AAD staining.

### Cell cycle analysis

Cell cycle analysis was performed with PI and flow cytometry. TNBC cells were plated in 6-well plates (3 × 10^5^ to 5 × 10^5^ cells per well) and treated with either SG, Apo2L or combination. Cells were harvested after 48–72 h, then washed twice with PBS and fixed in 70% ethanol for at least 1 h at 4 °C. Cells were then washed twice with cold PBS and resuspended in 50 µl of 100 µg/mL RNase A and 200 µl of 50 µg/ml PI. After a 1-hour incubation at room temperature, cell cycle was analyzed on a BD LSRFortessa™ flow cytometer and the data was analyzed using FlowJo^®^ software.

### Alkaline comet assay

Cells were seeded in 6-well plates (2-3 × 10^5^ cells/well) and treated with SG and/or Apo2L for 48 h. Damaged DNA was measured by alkaline comet assay using CometSlide™ High throughput slides (20 samples /slide) according to the manufacturer’s instructions (cat# 4252 − 500, R&D systems). Processed slides were stained with SYBR green 1 nucleic acid stain (cat# S7563, Thermo Fisher Scientific) in TE (10 mM Tris-HCL, 1 mM EDTA, pH 8.0) buffer for 3 min, washed in running tap water for 10 min, followed by a brief wash in deionized water. Slides were air dried for 15 min at 37 °C and imaged with a Nikon TS2 inverted fluorescent microscope. Tail moments indicative of damaged DNA were measured in 100–300 cells/condition with CometScore Pro (TriTek Corporation, Sumerduck, VA). Three independent experiments were performed for each cell line.

### Immunoblotting

Cells lysates were collected from TNBC cells and immunoblotting was performed as previously described [39]. Anti-Trop2 (cat# 90540 S), anti-PARP (#9542), anti-cleaved PARP (#5625), anti-caspase 3 (#9662), anti-cleaved caspase 3 (#9661), anti-cyclin B1 (#12231), and anti-γH2AX (#9718) antibodies were purchased from Cell Signaling Technology (Danvers, MA). Anti-DR4 (cat# GTX28414, GeneTex, Irvine, CA), anti-DR5 (cat# sc-65314, Santa Cruz, Dallas, TX), HSC70 (cat# sc-7298, Santa Cruz Biotechnology, Dallas, TX) and phospho-histone H3 (cat# ab136810, Abcam, Cambridge, UK) were also used. Goat Anti-Mouse IgG HRP conjugate (cat# 1721011) and Goat Anti-Rabbit IgG HRP conjugate (cat# 1721019) were purchased from Bio-Rad (Hercules, CA).

### Animal studies

All animal experiments were approved by the National Cancer Institute Animal Care and Use Committee (ACUC) and performed in compliance with all relevant ethical regulations and guidelines. To investigate the effects of SG and TRAIL agonist (Apo2L or MEDI3039) combination treatment on tumor growth and survival, 4-8-week-old immunocompromised NCr athymic nude (nu/nu) female mice were utilized. 4 × 10^6^ HCC1806 cells suspended in 100 µl of 1:1 matrigel (Corning, cat# 356237):PBS mixture were injected in the mammary fat pad (MFP).

For Experiment 1, average tumor size was allowed to grow to 150–200 mm^3^ after which mice were randomized into four treatment groups (10 mice per group): control (PBS), SG (100 ug), TRAIL/Apo2L (100 ug) and combination. SG was administered once a week intraperitonially on day 1 while Apo2L was administered three times a week intraperitonially on days 2–4. Due to limited availability of Apo2L, treatment was ended after two weeks versus the intended 5 weeks. Disease burden and body weight continued to be monitored twice a week. Tumor volume was calculated by using the Eq. 0.52 x longest tumor dimension ×shortest tumor dimention^2^.

Experiment 2 was conducted to test the effects of combination treatment with SG and TRAIL agonist for a full treatment regimen of 5 weeks. The experiment utilized TRAIL agonist MEDI3039, which has an improved half-life in mice (15.4 h) compared to Apo2L (3–5 min) [26, 40]. 4 × 10^6^ HCC1806 cells were injected in the MFP of athymic female mice. Once the tumor reached average size of 50–100 mm^3^, mice were then randomized to 4 groups (15 mice per group): control (PBS), SG (100 ug), MEDI3039 (0.1 mg/kg), and combination groups where each group had similar average baseline tumor size. SG was administered intraperitonially once a week on day 1 and MEDI3039 was administered the following day via tail vein injection (0.20 ml/23 g body weight). The treatments continued for five weeks and tumor growth and body weight were monitored twice a week. After two weeks of treatment, tumors were harvested from 3 to 5 mice from each experimental group. Tumor histology was analyzed as described below and protein expression was also assessed by Western blot. Protein lysates from sections of murine tumor samples were prepared using a tissue extraction reagent (cat# FNN0071, Invitrogen). For the remaining 10 mice per group, tumor size and survival continued to be monitored.

Once tumor size reached 20 mm in a single dimension, animals were euthanized per the NCI ACUC regulations. Drug toxicity was monitored where any mouse with a 15% or more drop in weight from time of tumor injection was euthanized. HCC1806 xenograft tumors have previously been found to have a propensity for poor vascularization and a tendency to develop tumor necrosis even without drug treatment [41]. Mice were euthanized if tumor necrosis and ulceration occurred in more than 50% of the tumor surface area.

### Histopathology

Tumor samples harvested from mice were embedded in optimal cutting temperature (OCT) compound and kept frozen at -80 °C. Tumor sections from the OCT frozen tissues were generated for H&E and immunohistochemistry (IHC) for cleaved caspase 3 (cat# 9661, Cell Signaling). Whole slide images were created using Aperio AT2 scanner with 20x objective. Positive and negative controls were stained appropriately for all markers. Tumor sections were annotated and staining was quantified using image analysis (Indica Labs, HALO) in annotated regions containing viable tumor. Normal adjacent tissues, histologic artifact, and regions of central tumor necrosis were excluded from analyses.

### Statistical analysis

Student’s *t*-test was used to determine significant differences between two groups. One-way and two-way analysis of variance (ANOVA) was also performed where indicated. Synergy scores for combination studies were calculated using SynergyFinder [37]. Survival analyses for our in vivo studies were performed using the Kaplan–Meier method, and curves were analyzed with log-rank (Mantel–Cox) test. For all data shown, p values of < 0.05 were considered significant.

## Results

### Trop2 expression varies among TNBC cell lines

**Fig. 1:**
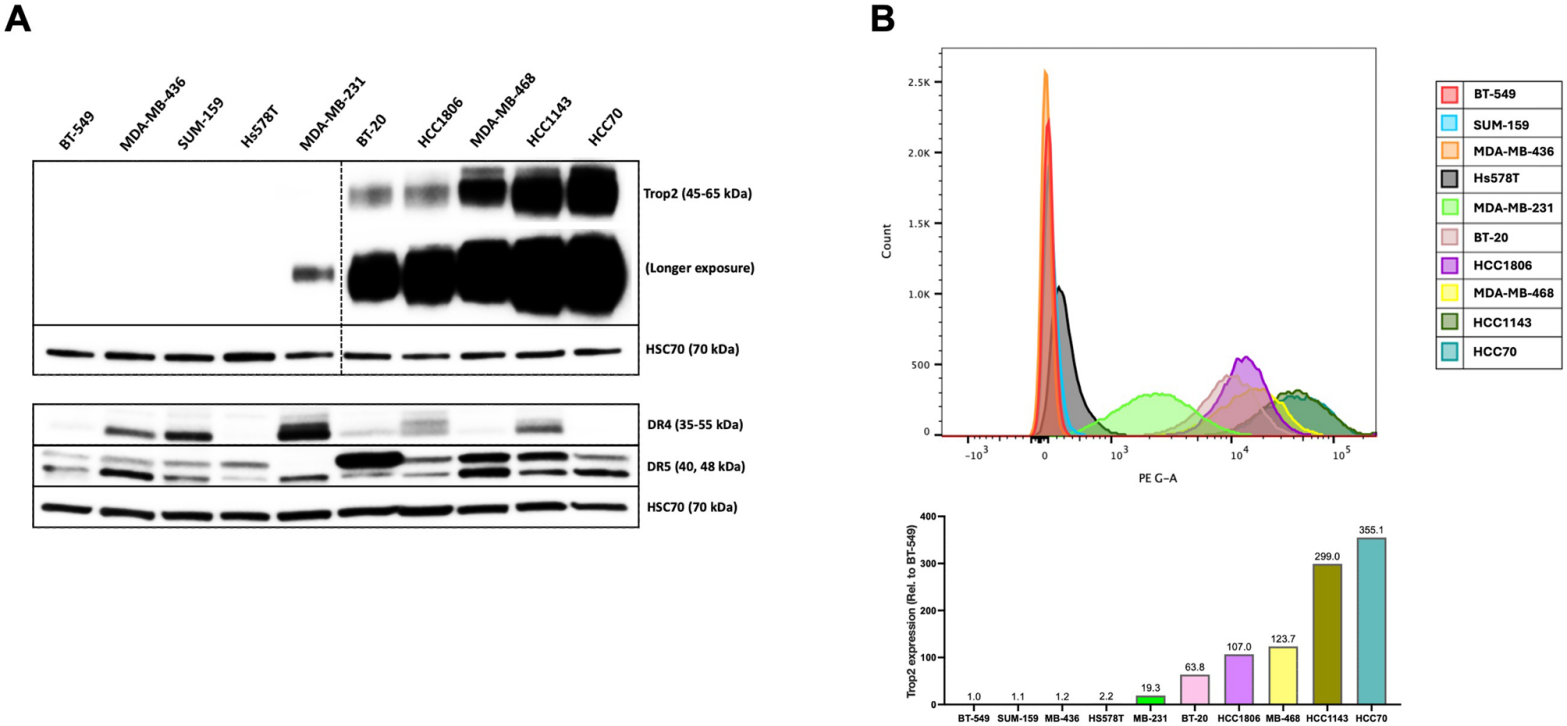
Trop2, DR4 and DR5 expression in TNBC cell lines. **A** Trop2, DR4 and DR5 expression measured by immunoblotting in TNBC cell lines. For the Western blot measuring Trop2, two blank lanes were used between low Trop2 expressor MDA-MB-231 and higher Trop2 expressor BT-20 to prevent the signal from BT-20 obscuring the signal in MDA-MB-231 lane during immunoblotting. As indicated by the dashed line, these lanes have been cropped out to fit the figure. **B** Trop2 expression assessed by flow cytometry. Trop2 molecules per cell were calculated using QuantiBRITE PE beads and graphed relative to the lowest expressor BT-549, which has 371+/- 32 receptors per cell. Bar graph data is presented as mean of three independent assays

To determine baseline level of Trop2 protein expression in our model TNBC cells, lysates from 10 different TNBC cell lines were collected and immunoblotted (Fig. 1A). BT-549, MDA-MB-436, SUM-159, Hs578T and MDA-MB-231 had little to no detection of Trop2. In MDA-MB-231, the Trop2 expression band was only visible with longer exposure at 30 µg loaded protein. Comparatively, BT-20, HCC1806 and MDA-MB-468 cells had medium expression levels of Trop2, while HCC1143 and HCC70s were the highest expressors. When protein loaded into gels was increased to 40 µg and captured with longer exposure, a faint Trop2 band was detected for Hs578T (Additional file 1). The Western blot data was corroborated by surface expression of Trop2 as evaluated by flow cytometry (Fig. 1B). The bar graph in Fig. 1B summarizes Trop2 expression across the multiple cell lines relative to BT-549, which exhibited the lowest Trop2 expression (371 +/- 32 estimated molecules per cell). It is important to note that the Western blot and flow cytometry-based assays can detect Trop2 expression in Trop2 positive cells but are not sensitive enough to differentiate between very low versus no Trop2 expression. Cell lines that have no detectable Trop2 band on Western blot and are estimated to have very low Trop2 molecules per cell by flow cytometry (i.e., BT-549, MDA-MB-436 and SUM-159) may or may not express Trop2.

### Trop2-directed antibody drug conjugate SG and the TRAIL agonist Apo2L synergistically induce TNBC cell death

**Fig. 2:**
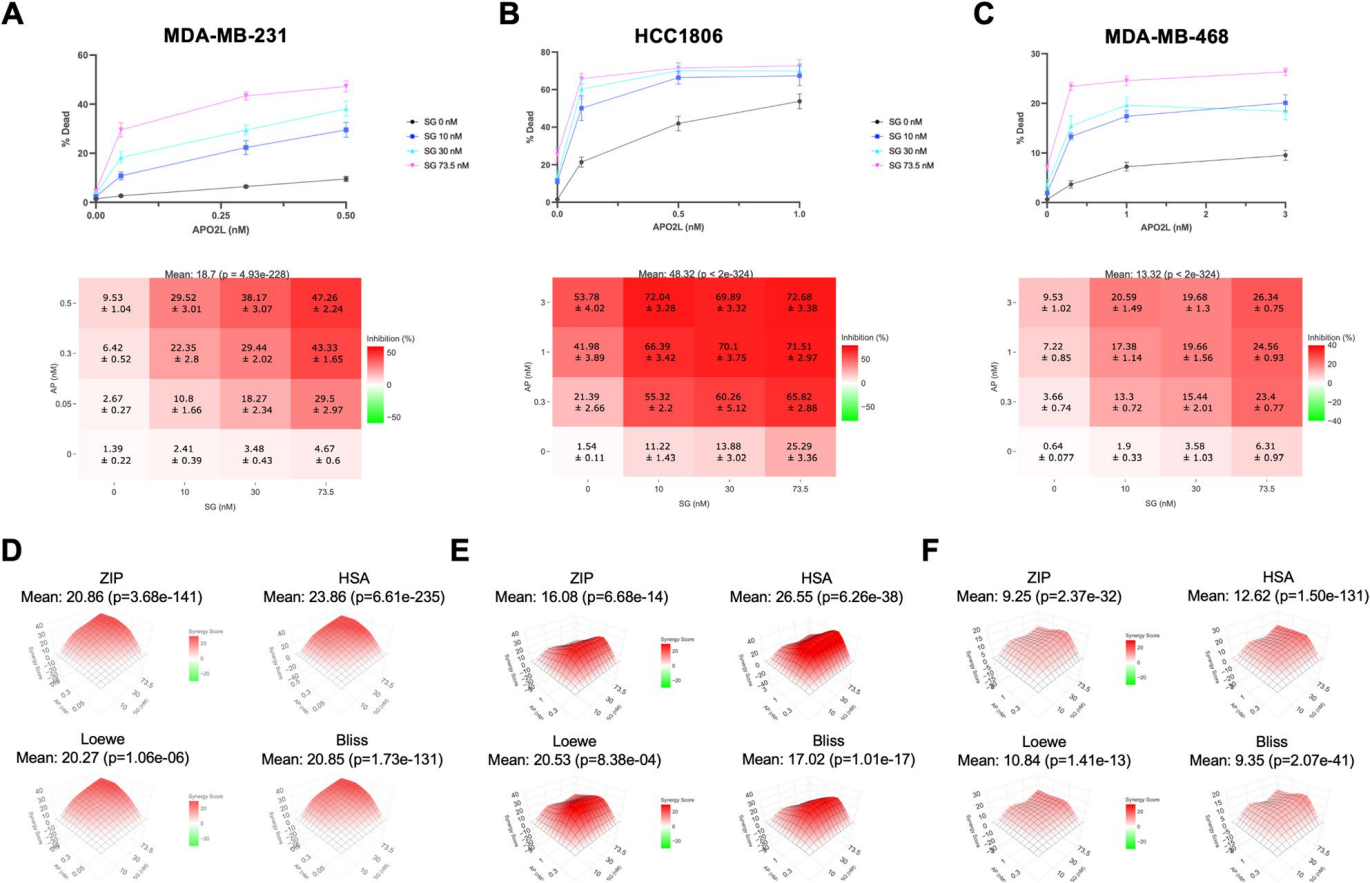
Trop2-directed antibody drug conjugate (SG) and TRAIL agonist Apo2L synergistically induce TNBC cell death. **A**, **B**, **C** HCC1806, MDA-MB-231 and MDA-MB-468 TNBC cells were treated with SG and Apo2L. Cell death was analyzed 48 h later using a propidium iodide-based cell death assay that was read on BioTek Cytation 1. Combination treatment with the two drugs synergistically induced cell death. Data represents the average death (± SEM) of three independent experiments. The top graph shows the data for each cell line and the bottom heat map shows the synergy matrix. D, E, F) Synergy scores and surface maps of repeat experiments were calculated using SynergyFinder. A synergy score of larger than 10 is likely synergistic; from − 10 to 10 is likely additive; less than − 10 is likely antagonistic

The effects of SG and Apo2L combination treatment on cell death was examined using a propidium iodide-based assay. Initially, 3 cell lines with variable baseline levels of Trop2 as well as a range of sensitivities to SG and Apo2L were evaluated (Fig. 2A-C). Dual treatment with SG and Apo2L conferred synergistic lethality in HCC1806 and MDA-MB-468 (medium Trop 2 expression) as well as MDA-MB-231 (low Trop2 expression) where cell death was synergistically enhanced compared to control and single treatment of each drug (Fig. 2D-F). Calculations by SynergyFinder showed scores of approximately 10 or greater using four different models (ZIP, Loewe, HAS and Bliss) indicating a synergistic effect with combination treatment [37]. We expanded our studies to 7 additional TNBC cell lines with variable Trop2 levels and drug sensitivities, which also showed similar results (Table 1, Additional file 2). CellTiter Glo 2.0 ATP viability assay was utilized for cell lines where computation of cell death using the Cytation 1 cell imager was challenging because of cell morphology differences (HCC70, HCC1143, BT-20 and Hs578T). For all 10 TNBC cell lines, combination treatment with SG and Apo2L synergistically induced death or reduced TNBC cell viability (Table 1). For MDA-MB-468 and MDA-MB-436, CellTiter-Glo 2.0 cell viability assays were also conducted in addition to cell death assays, both showing similar trends of drug synergy (Additional file 3). To evaluate the role of the SG bound SN-38 cytotoxic payload in the synergy observed, we used equimolar concentrations of free SG and tested combination effects with Apo2L. The data shows that dual treatment with SN-38 and Apo2L synergistically kills MDA-MB-231 (Additional File 4 A, C) and HCC1806 cells (Additional File 4B, D).

**Table 1:** Average synergy scores for combination treatment with sacituzumab govitecan and TRAIL agonist Apo2L

Cell lines	Trop2 surface expression*	ZIP	Loewe	HSA	Bliss
HCC70	131,752 ± 35,644	28.99	28.06	30.42	31.55
HCC1143	110,950 ± 4163	13.69	14.52	14.7	13.95
MDA-MB-468	45,891 ± 3977	9.25	10.84	12.62	9.35
HCC1806	39,691 ± 7335	16.08	20.53	26.55	17.02
BT-20	23,685 ± 4285	16.86	17.75	29.08	15.90
MDA-MB-231	7147 ± 1233	20.86	20.27	23.86	20.85
Hs578T	821 ± 141	27.94	8.81	18.52	29.54
MDA-MB-436	429 ± 20	16.07	13.43	17.74	16.37
SUM-159	405 ± 139	17.07	21.65	23.86	20.64
BT-549	371 ± 32	17.70	29.54	31.72	17.79

Cell viability was assessed after either 24–48 h of drug treatment using propidium iodide-based cell death assay or CellTiter-Glo 2.0 ATP-based cell viability assay (for HCC1143, HCC70, BT-20 and Hs578T cell lines). Trop2 molecules per cell were estimated via flow cytometry and estimated using QuantiBRITE PE beads. *Mean ± SD Trop-2 surface molecules per cell calculated by flow cytometry from three separate assessments. Synergy scores of at least three repeat experiments were calculated using Synergyfinder. A synergy score of larger than 10 is likely synergistic; from − 10 to 10 is likely additive; less than − 10 is likely antagonistic

Strong synergistic effect between SG and Apo2L were detected even in cells with low expression of Trop2 such as BT-549 and Hs578T. We hypothesized that extracellular linker deconjugation and bystander effect is in part responsible for the efficacy of SG and its synergy with Apo2L observed in Trop2 low cells. To test this hypothesis, BT-549 cells (low Trop2) and HCC1806 (mid Trop2) were preincubated with SG for 4 h (compared to continuous treatment for 24–48 h); afterwards, the media containing SG was removed and Apo2L was added. Of note, these two cell lines showed similar sensitivity to free SN-38 (Additional File 5 A). The modified protocol minimizing potential for linker deconjugation showed that a synergistic lethality with SG and Apo2L treatment remained in HCC1806 cells but was no longer observed in BT-549 cells (Additional File 5B). This suggests that the synergy observed in the Trop2 low cells is likely due to linker deconjugation.

### Combination of SG and Apo2L induces cell death via caspase dependent apoptosis

**Fig. 3:**
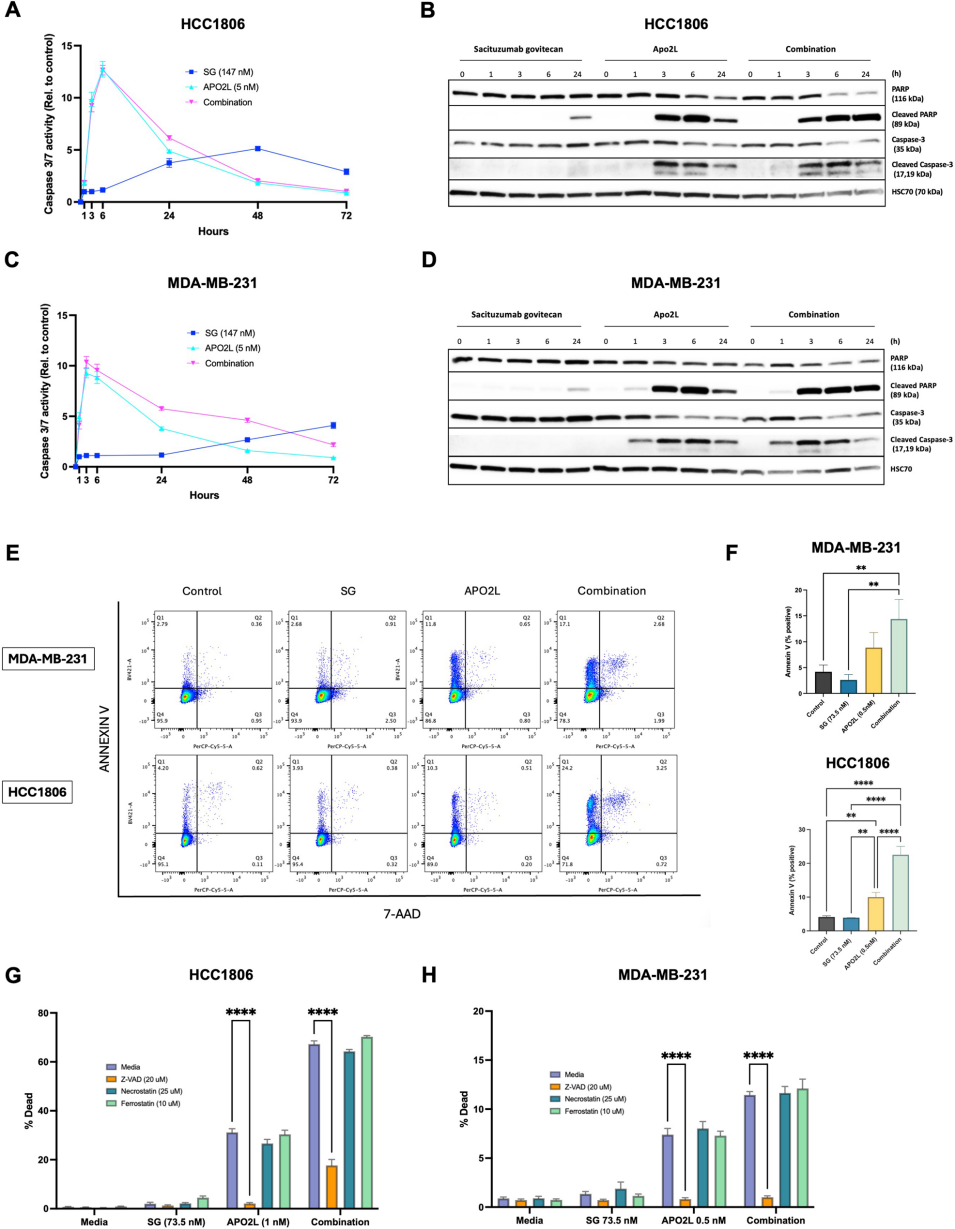
SG and Apo2L treatment cause cell death through caspase-dependent apoptosis. **A**, **C** HCC1806 and MDA-MB-231 cells were treated with SG and Apo2L for 1, 3, 6, 24, 48 and 72 h, respectively. Caspase activation was assessed with a Promega Caspase 3/7 assay. **B**, **D** Western blot showing time-dependent effect of SG and Apo2L in HCC1806 (30 nM SG; 1 nM Apo2L) and MDA-MB-231 (73.5 nM SG; 0.7 nM Apo2L), respectively. **E** Plots of flow cytometry-based annexin V/7-AAD analysis showing fractions of apoptotic cells in MDA-MB-231 and HCC1806 cells. **F** Quantification of percentages of apoptotic cells in each treatment condition. **G**, **H** Cells were pretreated with cell death inhibitors Z-VAD-FMK, necrostatin and ferrostatin for one hour after which SG and Apo2L drugs were added for 24 h to assess cell death rescue. Cell death was measured by propidium iodide-based cell death assay. Statistical significance was determined using Student’s *t*-test. * indicates *p* < 0.05, ** indicates *p* < 0.01 and *** indicates *p* < 0.001, **** indicates *p* < 0.0001. Data is shown as average ± SEM of multiple independent experiments (*N* ≥ 3)

HCC1806 and MDA-MB-231 cells were treated with SG and Apo2L after which caspase 3/7 activation was measured over time (Fig. 3A, C; Table S1). Apo2L single drug treatment caused a significant (≥ 10-fold) increase in caspase 3/7 activity in as early as 3 h and 4 h in both cell lines. SG was slower acting at the dose given with a ≥ 5-fold increase in activation occurring at 48 h for HCC1806 and 72 h for MDA-MB-231. Notably, dual treatment with SG and Apo2L had greater caspase 3/7 activation compared to either drug alone for MDA-MB-231 from 3 h to 48 h. This increase was only briefly observed at the 24 h timepoint in HCC1806 cells, most probably because of the high lethality of the combination treatment in this cell line, leading to only a few viable cells after 24 h. To further quantify the caspase 3/7 activity induced by the combination compared to the individual drugs, we calculated the area under the curve for caspase 3/7 activation over time, which indicated significant increase of caspase 3/7 activation in the combination treatment condition compared to either drug alone in MDA-MB-231 cells and compared to SG in HCC1806 cells (Table S1). Additionally, immunoblotting indicated that biochemical markers of caspase-dependent apoptosis, such as cleaved caspase 3 and cleaved PARP were increased with Apo2L and at later timepoints, SG (Fig. 3B, D). To quantify apoptosis induction on a per cell basis, flow cytometry-based annexin V/7-AAD staining was employed and results confirmed that combination treatment with SG and Apo2L significantly increased the fraction of annexin V positive/7-AAD negative cells, consistent with enhanced induction of apoptosis in MDA-MB-231 and HCC1806 cells (Fig. 3E, F). Moreover, cell death was assessed with combination treatment by PI uptake in the presence or absence of multiple cell death inhibitors including caspase inhibitor (Z-VAD-FMK), ferroptosis inhibitor (ferrostatin) and necroptosis inhibitor (necrostatin). Apo2L alone induced significant cell death that was rescued by Z-VAD-FMK but not the other inhibitors. Meanwhile, SG alone did not induce significant cell death compared to controls at 24 h. Dual treatment with SG and Apo2L caused a significant increase of dead cells compared to either drug alone, and this was rescued by Z-VAD-FMK treatment, while necrostatin and ferrostatin did not rescue cell death (Fig. 3G and H). Together, these data indicate that caspase-mediated apoptosis is the predominant mechanism of cell death caused by the combination of SG and Apo2L.

### DNA damaging agent carboplatin and the TRAIL agonist Apo2L synergistically induce TNBC cell death via caspase dependent apoptosis

To determine if our findings can be generalized to other DNA damaging cytotoxic agents aside from SG, we tested combination treatment with DNA alkylating agent carboplatin and Apo2L. MDA-MB-231 and HCC1806 cells were treated with both drugs and cell death was assessed 48 h later. Strong synergistic effect between carboplatin and Apo2L was found in both cell lines with high synergy scores (Additional File 6 A-D). Rescue assays with cell death inhibitors also demonstrated that cell death due to combination treatment was only rescued by Z-VAD-FMK treatment, while necrostatin and ferrostatin did not rescue cell death, suggesting that caspase-dependent apoptosis is the primary mechanism of cell death involved (Additional File 6E, F).

#### SG induces increases in DR4 and DR5 surface expression

**Fig. 4:**
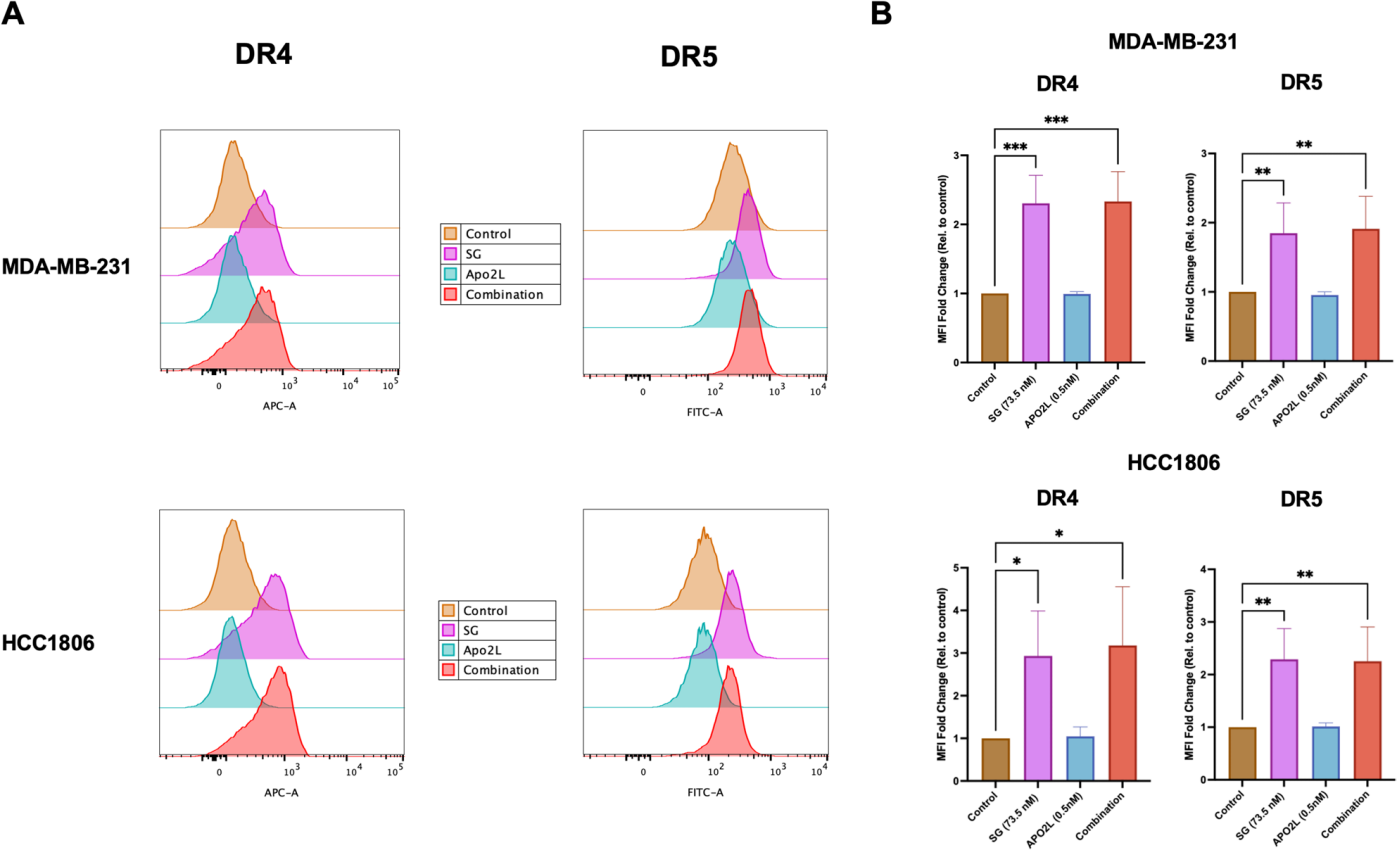
SG increases the expression of DR4 and DR5 receptors. **A** DR4 and DR5 surface expression was measured by flow cytometry 48 h after treatment with SG and Apo2L (a representative flow histogram is shown). **B** Median fluorescence intensity of receptor expression was calculated relative to controls. Statistical significance was determined using Student’s *t*-test. * indicates *p* < 0.05, ** indicates *p* < 0.01 and *** indicates *p* < 0.001, **** indicates *p* < 0.0001. Data is shown as average ± SEM of multiple independent experiments (*N* ≥ 3)

Previous work has shown that chemotherapeutic agents including DNA and microtubule disrupting agents can increase DR4/5 expression [31, 42, 43]. To test a similar potential effect by SG, we measured DR4 and DR5 expression after treatment with SG and Apo2L in MDA-MB-231 and HCC1806 cells. The data showed that surface expression of both DR4 and DR5 increased with SG and combination treatments while no effect was found for Apo2L single drug treatment (Fig. 4A). Combination treatment did not significantly enhance DR4/5 expression compared to SG alone, suggesting that this is an SG-driven effect. This effect led to an approximately 2–3-fold increase in DR4 and DR5 expression, suggesting a potential mechanism of synergy with Apo2L (Fig. 4B).

### SG induces DNA damage and cell cycle arrest at the G2/M phase

**Fig. 5:**
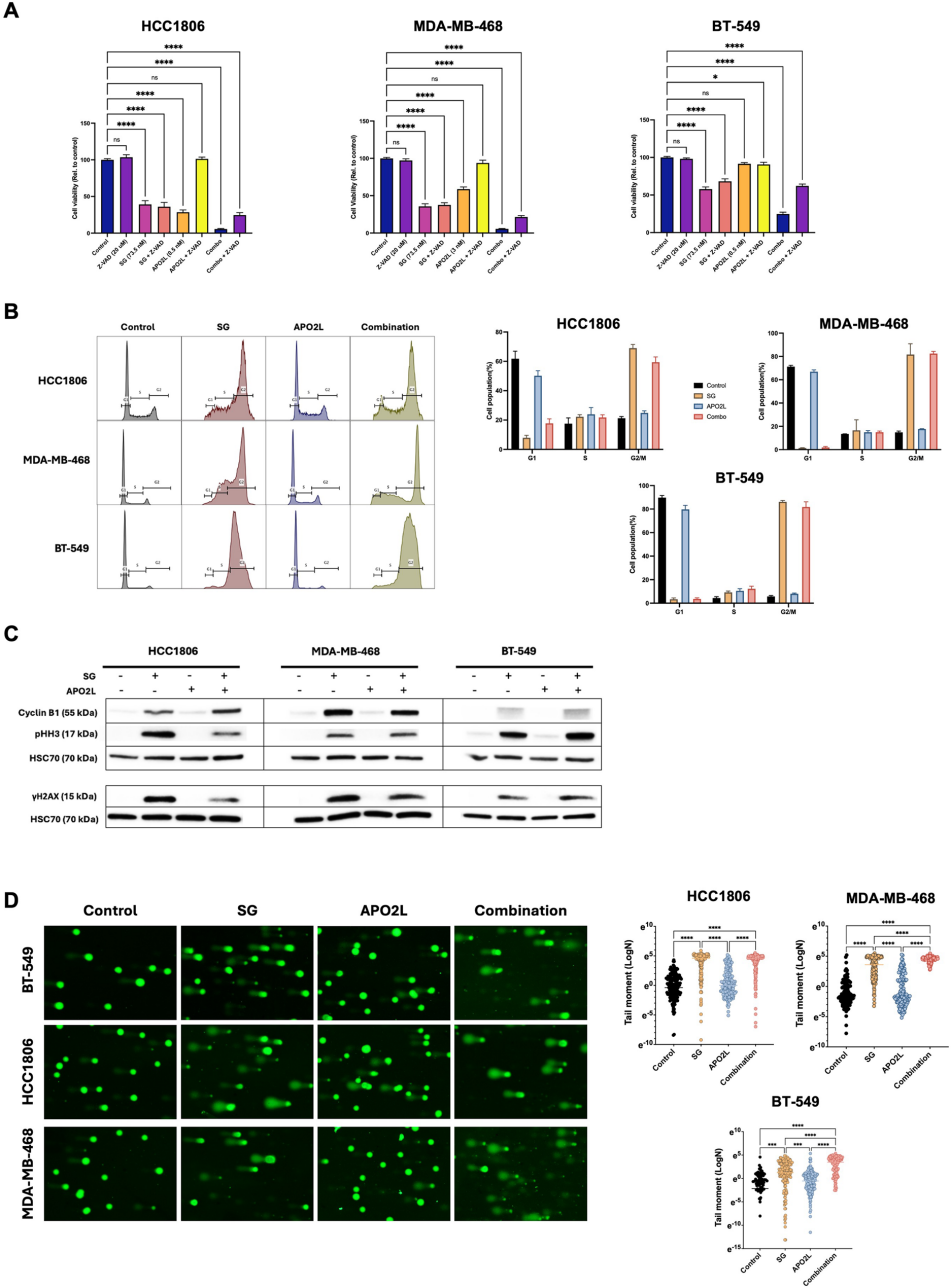
SG causes G2/M arrest in TNBC cells. **A** TNBC cells were pretreated for 1 hour with pan-caspase inhibitor Z-VAD-FMK after which they were treated with SG, Apo2L or combination for 48 hours. Cell viability was assessed using Promega CellTiter-Glo ATP cell viability assay to determine rescue of cell viability with ZVAD pre-treatment. **B** Flow cytometry-based cell cycle analysis was conducted after treating cells with 30 nM SG and 0.1 nM Apo2L for 48 hours (HCC1806 and BT-549) or 73.5 nM SG and 1 nM Apo2L for 72 hours (MDA-MB-468). Bar graphs are representative of 3 biologically independent experiments with cell population percent +/- SEM. **C** Drug treated MDA-MB-231, MDA-MB-468 and BT-20 cells were lysed and proteins immunoblotted for markers of G2/M cell cycle arrest (cyclin B1 and phospho-histone H3 (pHH3)) as well as double stranded DNA damage marker γH2AX. **D** Representative images of alkaline comet assay performed on all three cell lines and scatter plots representative of at least three independent experiments. One-way ANOVA was conducted to determine significant differences compared to control. ns indicates not statistically significant, * indicates p<0.05, ** indicates p<0.01 and *** indicates p<0.001, **** indicates p<0.0001

Next, we tested the effect of SG and Apo2L treatment on cell viability in HCC1806, MDA-MB-468 and BT-549 cells using CellTiter Glo 2.0. In contrast to the aforementioned finding on cell death, cell viability was only partially rescued by Z-VAD-FMK for SG and combination treatment (Fig. 5A) in all three cell lines, while Apo2L mediated reduction in cell viability was fully rescued with Z-VAD. This suggested a possible anti-proliferative role of SG in addition to its observed effect on cell death. Cell cycle analysis using flow cytometry showed that SG alone and combination treatment with Apo2L had a significant increase in TNBC cells in the G2/M phase with concomitant decrease in G1 phase populations in all 3 TNBC cell lines tested; the G2/M accumulation in the combination was largely due to SG as no such accumulation in G2/M phase was observed with Apo2L alone nor was it increased in the combination condition (Fig. 5B). Consistent with these observations, SG and dual treatment induced expression of markers of G2/M cell cycle arrest including phospho-histone H3 and cyclin B1 in all three cell lines (Fig. 5C). Increase in γH2AX was also found with SG and combination conditions, suggesting SG-mediated increases in double stranded DNA damage. DNA strand breaks were identified using the alkaline comet assay, revealing a significant increase in tail moment with SG and combination treatments across all three cell lines (Fig. 5D). In MDA-MB-468 and BT-549 cells, the combination treatment caused even greater tail moments than SG alone. No DNA damage was observed with Apo2L treatment alone. Overall, these results suggest that SG causes DNA damage and leads to cell cycle arrest at the G2/M phase.

### Dual treatment induces synergistic lethality in patient-derived primary TNBC cells

**Fig. 6:**
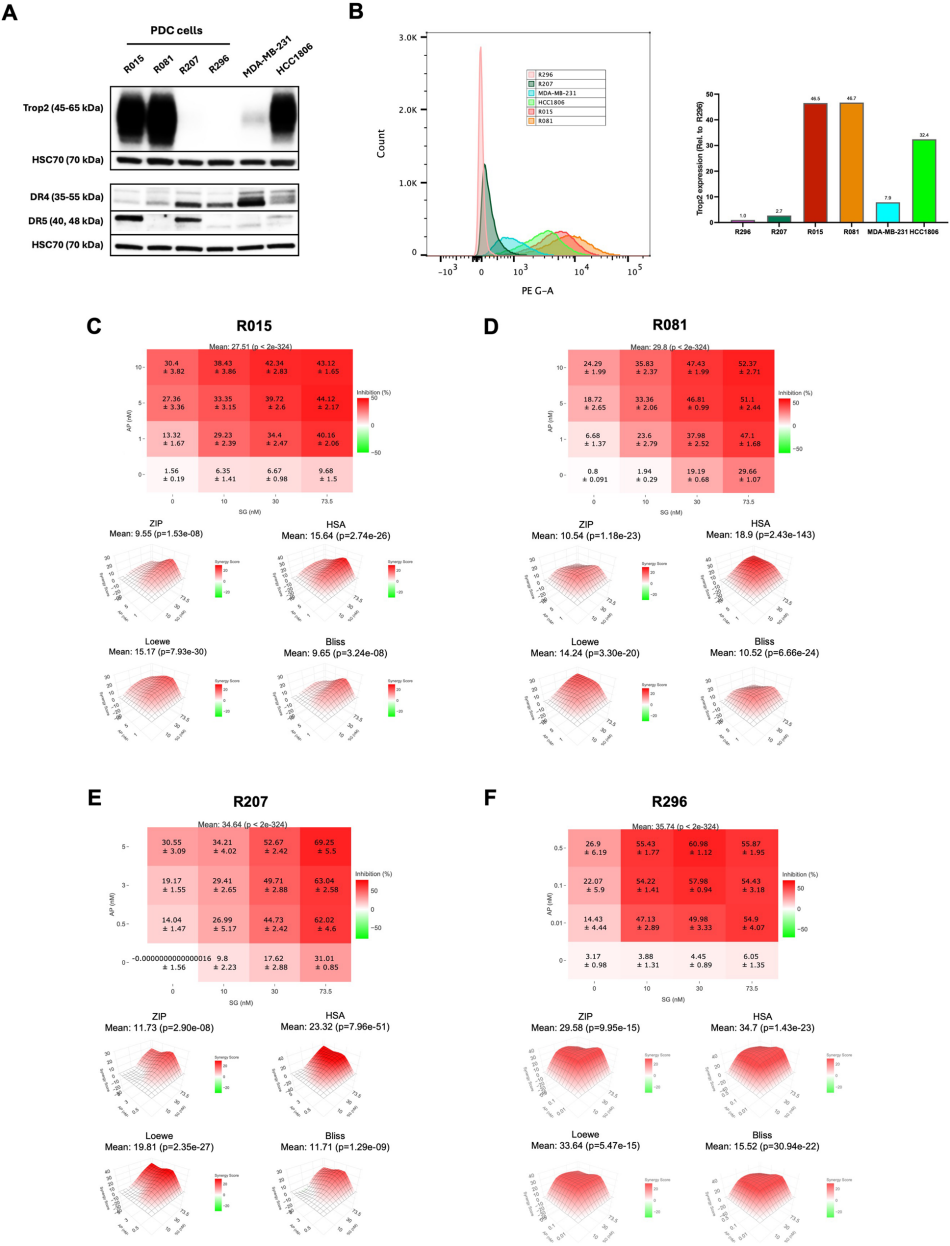
SG and Apo2L synergistically induce cell death in patient derived TNBC cells. **A** Trop2 expression in patient derived TNBC cells R015, R081, R207 and R296 as measured by immunoblotting. TNBC cell lines MDA-MB-231 and HCC1806 are included for comparison. **B** Trop2 surface expression assessed by flow cytometry in TNBC patient derived cells and cell lines. Surface Trop-2 molecules per cell were calculated using QuantiBRITE PE beads and graphed relative to the lowest expressor, R296 (402 +/- 10 receptors per cell). **C**, **D**, **E**, **F** Patient derived TNBC cells were treated with SG and Apo2L. Cell death was analyzed for R015, R081 and R296 at 24 h using a propidium iodide-based cell death assay. Cell viability was assessed for R207 at 48 h using Promega CellTiter-Glo ATP cell viability assay. Data represents the average death/viability (± SEM) of 3 independent experiments. Synergy scores of repeat experiments were calculated using SynergyFinder. A synergy score of larger than 10 is likely synergistic, from − 10 to 10 is likely additive, less than − 10 is likely antagonistic

We further examined the efficacy of SG and Apo2L combination treatment in genetically non-modified, low passage patient derived TNBC cells acquired from NCI PDMR. Baseline Trop2 levels of 4 different patient derived cells were first assessed. R207 and R296 had little to no detection of Trop2 by immunoblotting and flow cytometry, while R081 and R015 had higher expression of Trop2 (Fig. 6A-B). MDA-MB-231 and HCC1806 cells were included in the analysis for comparison demonstrating that the range of Trop2 expression seen in the PDC cells was similar to that seen in the TNBC cell lines. Cell death and cell viability assays showed that combination treatment of SG and Apo2L synergistically inhibit cells in all 4 PDC cells with high synergy scores (Fig. 6C-F, Additional File 7). These data demonstrate that primary cells derived from TNBC patients respond similarly to SG and Apo2L compared with TNBC cell lines.

### ER+/HER2- breast cancer cells are synergistically inhibited by SG and Apo2L dual treatment

SG is approved for use in treatment refractory ER+/HER2- breast cancer [44]. We examined if the synergy between SG and Apo2L that was observed in TNBC can also be found in ER+/HER2- subtype of breast cancer cells. Baseline Trop2, DR4 and DR5 levels were initially assessed in MCF7 and T47D ER+/HER2- cells (Additional File 8A). The range of Trop2 expression seen in these cells was similar to that of TNBC cell line HCC1806. Cells were then treated with SG and Apo2L after which cell death was assessed for T47D using the Cytation 1 imager. ATP-based cell viability assay was utilized in MCF7 cells because cell morphology made computations of cell death by the Cytation 1 cell imager challenging. The results showed that dual treatment with SG and Apo2L synergistically inhibited both cells with high synergy scores (Additional File 8B-E).

### SG and TRAIL agonist combination inhibits tumor growth in vivo

Next, we tested the effect of dual treatment with SG and TRAIL agonist in a xenograft breast cancer model. Of note, the doses of SG and TRAIL agonist used in vivo were purposefully chosen to have only modest effects on tumor growth in order to better detect the combination effect. For the first experiment, SG and Apo2L were tested in HCC1806 tumor bearing mice (Additional File 9 A). As illustrated, SG was given weekly on day 1 of each week, and Apo2L was given subsequently on days 2–4. After two weeks of treatment, average tumor size was significantly decreased by the combination treatment compared to the control and both monotherapy groups (Day 29, *p* < 0.05; Additional File 9B, C), although the anti-tumor effect was no longer observed after the treatment was discontinued. The combination treatment was well tolerated as no significant change of body weight was observed (Additional File 9D).

**Fig. 7:**
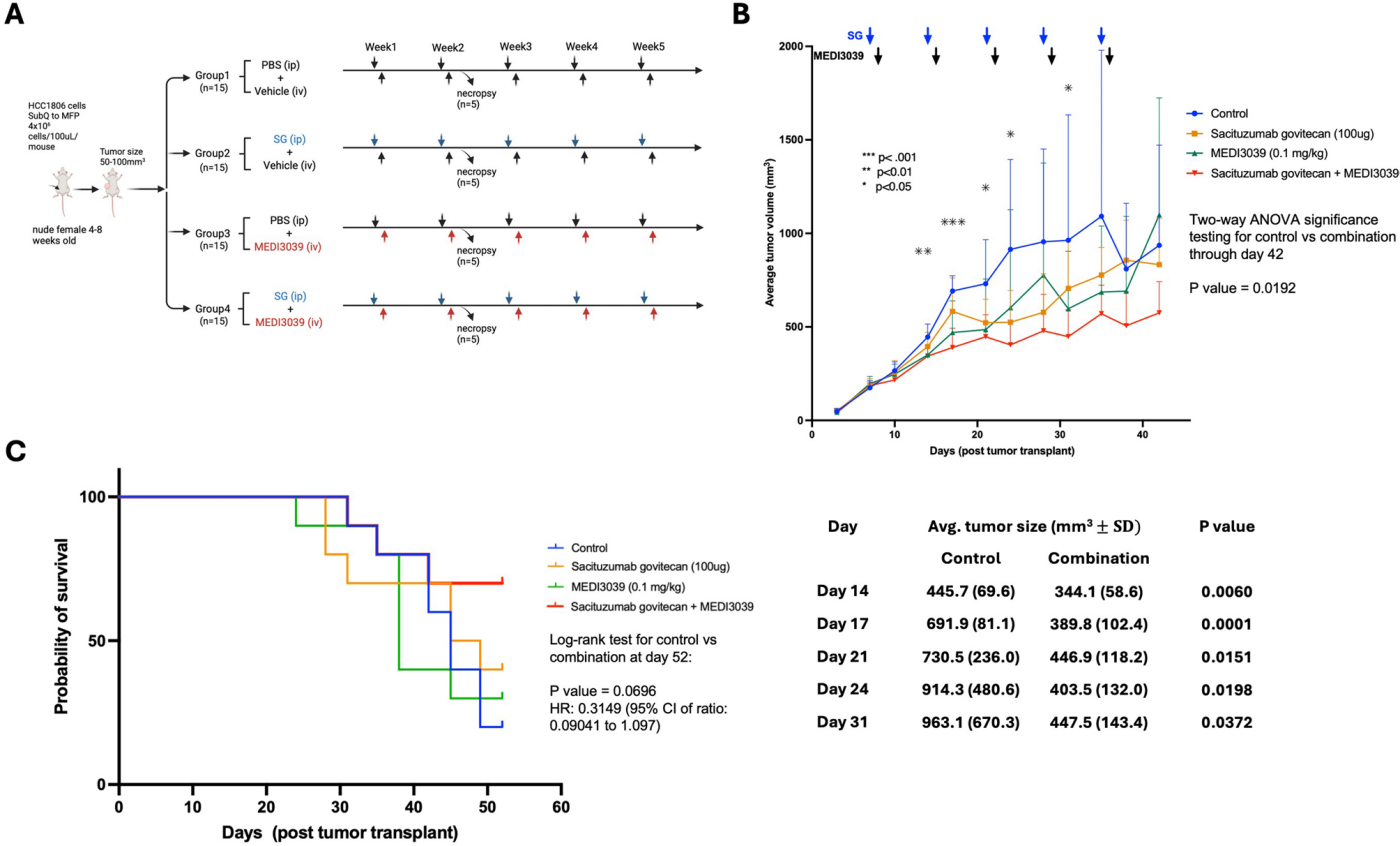
Dual treatment with SG and TRAIL agonist MEDI3039 reduces tumor growth in vivo*.* **A** In vivo experimental design. In athymic nu/nu nude mice bearing HCC1806 tumors, SG was administered once a week intraperitonially on day 1 while MEDI3039 was administered on day 2 once a week by tail vein injection at indicated doses. 5 mice from each group except combination (3 mice) were euthanized after 2 rounds of treatment (at day 16) for histopathology analyses. The remining 10 mice per group were followed for tumor growth and survival. **B** Tumor size was assessed twice a week. One-way ANOVA was conducted at each time point to compare significant differences between groups where significant differences between control and combination groups are marked by asterisks. Average tumor sizes and significance testing results are listed below. Two-way ANOVA was utilized to evaluate tumor growth differences between groups over time. Longitudinal analyses were performed up to day 42 because multiple mice from all treatment groups were developing significant ulcerations (including controls, unrelated to treatment) and prematurely getting euthanized after that timepoint, affecting average tumor size calculations. **C** Survival curve of mice treated with SG, and MEDI3039. Significance testing of differences in survival between groups was conducted using Log-rank test

Subsequently, we examined the combination therapy effect using a potent TRAIL agonist MEDI3039 with a longer half-life of ~ 15 h in mice compared to 3–5 min for Apo2L [26, 40]. In vitro studies using HCC1806 cells confirmed that MEDI3039 is at least two orders of magnitude more potent that Apo2L (picomolar range versus nanomolar range, respectively) (Fig. 2A, Additional File 10 A) as we had previously reported [26]. Additionally, combination of SG and MEDI3039 also showed synergistic lethality in the HCC1806 cell line in vitro (Additional File 10 A). In our second in vivo study using HCC1806 xenograft model, mice were treated with SG and MEDI3039 for 5 weeks with both SG given weekly on day 1 of each week and MEDI3039 given on day 2 as outlined in Fig. 7A. Dual treatment significantly inhibited tumor growth (Fig. 7B). Compared to control, combination treatment significantly inhibited tumor growth as early as one week after first treatment (day 14, *p* < 0.01) and the therapeutic effect of combination persisted for weeks (Fig. 7B; Table S2). Longitudinal analysis of tumor volume using two-way ANOVA showed that dual therapy significantly inhibited tumor growth compared to SG monotherapy (*p* = 0.0023) and the control (*p* = 0.0192) but the combination did not reach statistical significance when compared to MEDI3039 monotherapy (*p* = 0.1811) (Table S3). Furthermore, there was a borderline significant benefit on survival with dual treatment compared to control (*p* = 0.0696 with a HR = 0.319) (Fig. 7C). The median survival of the control group was 45 days, while that of the combination group did not reach a median survival at 52 days when the study ended. There were no significant effects on survival of the treatments with the single agents compared to control nor compared to the combination. Treatment with either drug alone or in combination was well tolerated with no significant reductions in mice body weight (Additional File 10B).

Of note, a previous study has shown that HCC1806 xenograft tumors tend to have poor vascularization and develop tumor necrosis even without drug treatment [41]. Consistent with this, we observed that HCC1806 tumors grown in mice often developed deep and large ulcerations regardless of treatment status, leading to large variability in the measurements and premature euthanasia before the tumor size threshold was reached. Therefore, further longitudinal analyses were not conducted.

### Dual SG and TRAIL agonist treatment increases tumor markers of apoptosis, DNA damage and cell cycle arrest

**Fig. 8:**
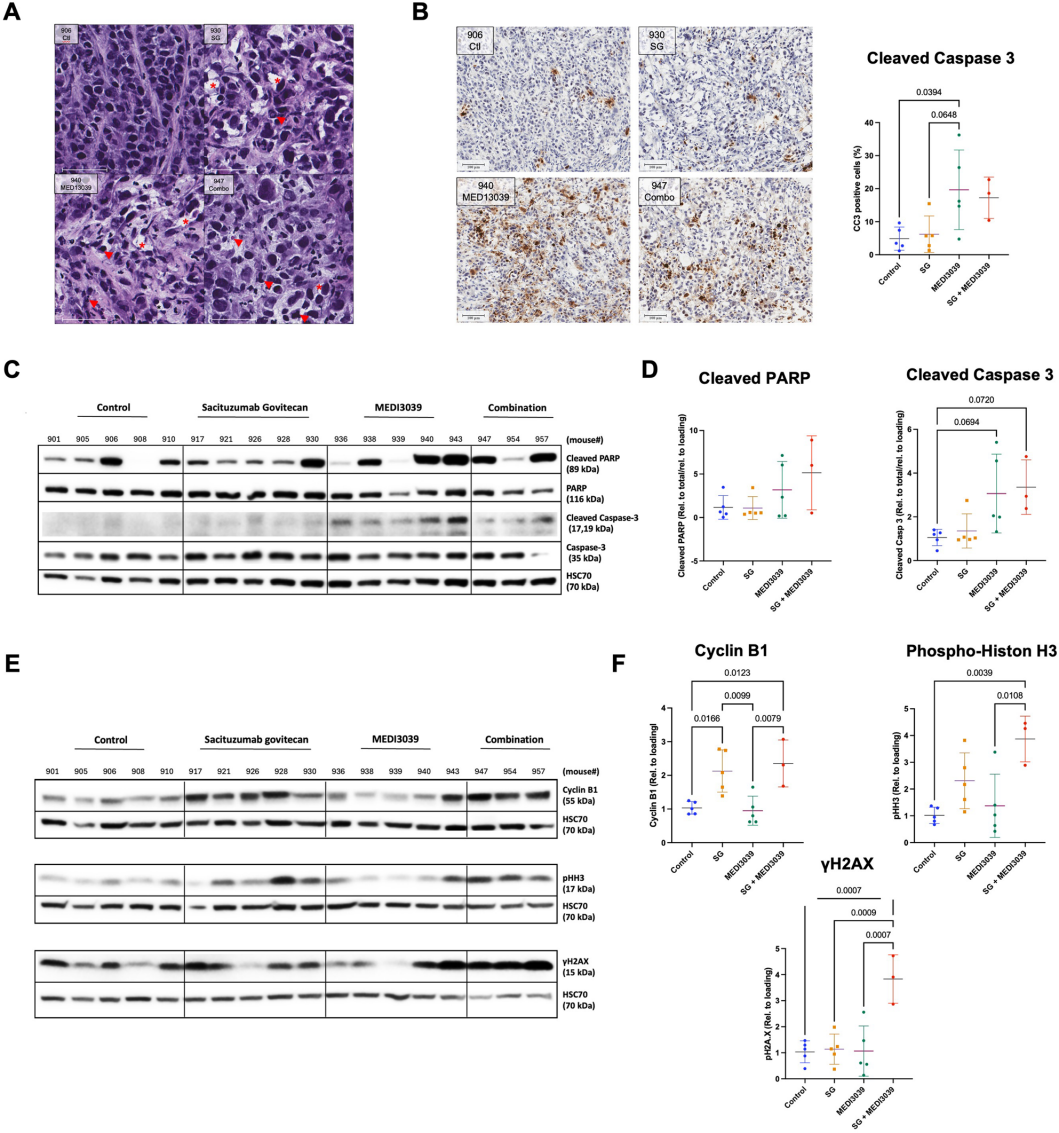
Increased biomarkers of apoptosis, DNA damage and G2/M arrest in SG and MEDI3039 treated tumors. One day after the second treatment, 3-5 mice from each group were euthanized and tumors isolated for microscopic evaluation and protein evaluation. **A** Representative hematoxylin and eosin (H&E) staining of tumor samples obtained after two rounds of treatment with SG and MEDI3039 (day 16). Tumors from 5 mice per group were obtained for control, SG and MEDI3039 groups while 3 mice per group were euthanized from the combination group. Compared to control tumors, treated sections contain cells with increased cytoplasmic vacuolation (*) and peripheralized nuclear profiles and cells with morphologic features consistent with apoptosis, including shrunken or fragmented nuclei (arrowhead). Scale bar 50um. **B** Immunohistochemistry analysis of tumors stained with cleaved caspase 3. Cells are quantified in viable tumor regions and reported as the number of positive cells per mm2 and the percentage of total cells (average ± SD). **C, D** Protein from tumors was isolated. Markers of apoptosis (cleaved caspase 3 and cleaved PARP), and **E, F** markers of G2/M cell cycle arrest (cyclin B1, phospho-histone H (pHH3)) and double stranded DNA damage marker γH2AX were immunoblotted. Data is presented as relative gene expression (average ± SD). Statistical analyses were conducted using Student’s t-test

To examine the pharmacodynamic drug efficacy on tumors, a few mice from each group of in vivo experiment 2 (Fig. 7A) were euthanized one day after the second treatment (day 16). Five mice were sacrificed from the control, SG and MEDI3039 groups while three mice were sacrificed from the combination group. Two other mice from the combination group had to be excluded early in the study due to non-study related technical errors. H&E analysis suggested a prevalence of cells with morphologic changes consistent with apoptosis observed in tumors treated with SG and MEDI3039 alone and in combination (Fig. 8A). Immunohistochemical quantifications of the apoptosis biomarker cleaved caspase 3 also showed increased percent of positive cells in MEDI3039 and combination conditions, although statistical significance compared to the control tumors was only observed in the MEDI3039 monotherapy group (*p* = 0.0392; Fig. 8B). Immunoblotting using tumor lysates showed significant increases in cleaved caspase 3 in MEDI3039 and combination treated tumors compared with the control group (Fig. 8C, D). Cleaved PARP was increased in the MEDI3039 and combination treated tumors but this did not reach statistical significance. In addition, cyclin B1 expression was significantly increased with SG and with combination treatment (*p* < 0.05), suggesting cell accumulation in the G2/M phase in these tumors (Fig. 8E, F). While the other G2/M arrest maker phospho-histone H3 was elevated in both SG alone and in the combination treatment conditions, it only reached significance with dual treatment (Fig. 8E, F). Lastly, γH2AX was significantly elevated in tumors treated with both SG and MEDI3039 (Fig. 8E, F).

## Discussion

New treatment approaches for TNBC include exploring novel drug combinations that synergistically inhibit cancer cells. In the present study, our in vitro data showed that treatment of TNBC with Trop2 directed antibody drug conjugate SG and TRAIL/Apo2L confers synergistic lethality against cancer cells including 10 TNBC cell lines with variable sensitivities to both drugs. Similar associations of synergy were found against four non-modified and low passage patient derived TNBC cells. Our in vivo studies utilizing HCC1806 bearing mice also demonstrated significant antitumor effects with combination treatment including inhibition of tumor growth as well as a borderline significant improvement in survival, suggesting a potential clinical benefit of combining SG and TRAIL agonists in TNBC.

Our data demonstrate that the mechanism of synergistic cell death with SG and Apo2L dual treatment in TNBC cells is caspase-dependent apoptosis. Previous studies have found that SG and its cytotoxic payload SN-38 (topoisomerase I inhibitor and irinotecan prodrug) cause double stranded DNA breaks and induce apoptotic cell death [19, 45–48]. TRAIL agonists’ mechanism of action entails binding death receptors 4 and 5 to activate the apoptosis pathway [49]. In this study, annexin V assays showed that dual treatment with SG and TRAIL significantly increases apoptotic cell death. In vitro studies also revealed that biomarkers of apoptosis (e.g., cleaved caspase 3 and cleaved PARP) were elevated with both SG and TRAIL single and combination treatment of cells, and cell death rescue assays with Z-VAD-FMK confirm that caspase-mediated apoptosis is the major mechanism of cell death. On the other hand, in vivo studies showed elevated expression of biomarkers of apoptosis only for tumors treated with TRAIL agonist MEDI3039 and combination, but not SG alone. This could be due to the relatively low dose of SG used in vivo, purposefully chosen to best observe the efficacy of combination treatment. These parameters are also time sensitive where their highest expression post drug treatment has to be identified, which is readily done in vitro but not in vivo.

In addition to cell death, our data demonstrated an antiproliferative activity for SG. Treatment with SG alone and in combination with Apo2L induced cell cycle arrest that is largely driven by SG in different TNBC cells. Alkaline comet assays and flow cytometry-based cell cycle analysis of HCC1806, MDA-MB-468 and BT-549 found that SG treatment increased DNA strand damage and caused an accumulation of cells in the G2/M phase with a concomitant decrease in the G1 phase. Furthermore, tumor lysate samples from our in vivo study showed an elevation in protein biomarkers of double stranded DNA damage and G2/M cell cycle arrest with SG monotherapy and combination therapy (Figs. 7F and 8E). The sample size for evaluating these tumor markers were small and unbalanced especially in the combination group. This limits our ability to make confident inferences from well powered associations. Yet, the trends observed are consistent with previous reports including in colorectal, ovarian and testicular cancer models demonstrating that SG and topoisomerase I inhibitors induce G2/M checkpoint arrest before the M phase transition [23, 50, 51]. These potential associations need to be further explored in additional xenograft studies with larger sample sizes.

In this study, the efficacy of SG as a single treatment and its synergistic interaction with Apo2L was observed in vitro in TNBC cells with both Trop2 high and Trop2 low cells (Fig. 2; Table 1). This finding is in accordance with previous studies showing that while higher Trop2 expression is associated with greater efficacy of SG [52], SG can also have substantial inhibitory effects in Trop2 low cells [18, 23, 52–54]. This is likely in part to be due to the extracellular linker deconjugation and bystander effects associated with ADCs including SG [55, 56]. In addition to binding to its Trop2 target and getting internalized in the cell, SG undergoes extracellular deconjugation of its hydrolysable linker and releases SN-38 into the extracellular milieu, allowing the cytotoxic payload to affect nearby cells including those with low Trop2 [12–18]. Furthermore, internalized SN-38 can also diffuse from one cell to another, further enhancing its efficacy in Trop2 low cells. Accordingly, our standard experiments with continuous exposure (24–48 h) of SG and Apo2L exhibited synergistic lethality for both HCC1806 (Trop2 mid) and BT-549 (Trop2 low) cells regardless of Trop2 level. On the other hand, when the exposure time of SG was shortened to only 4 h in order to minimize linker deconjugation and bystander effects, only Trop2 mid HCC1806 cells remained synergistic while Trop2 low BT-549 cells were no longer synergistic despite having similar sensitivity to SN-38. Linker deconjugation and bystander effects have been previously reported in both in vivo and in vitro models of cancer including TNBC where SG has significant cytotoxic activity in both Trop2 high and Trop2 low models [18, 23, 52–54].

In our first in vivo experiment, decrease in tumor growth was observed with Apo2L and SG dual therapy during and shortly after treatment ended, despite the limited treatment period (Additional File 9). In this experiment, at a timepoint immediately after therapy ended, the combination of SG and Apo2L induced a significant reduction in tumor size compared to the control or either drug alone. The treatment with Apo2L was compromised by the short half-life of Apo2L in vivo (3–5 min) resulting in limited exposure of the tumors to the drug [40]. In the second in vivo experiment, we used MEDI3039, a more potent TRAIL agonist, which has a significantly longer half-life (15 h) compared to Apo2L [26, 40]. With 5 weeks of treatment, combination of SG and MEDI3039 significantly inhibited tumor growth compared to control as early as one week after the first treatment and persisted for weeks. Of note, the combination inhibited tumor growth significantly compared to SG but not compared to MEDI 3039 alone. A borderline significant survival benefit was also noted with dual therapy.

Our in vitro studies also demonstrated synergistic lethality between TRAIL and SG cytotoxic payload and irinotecan prodrug SN-38 in TNBC. This suggests that topoisomerase I inhibitor SN-38 plays a major role in the synergy observed with the ADC platform SG. While a potent cytotoxic drug, SN-38’s poor solubility and rapid glucuronidation in vivo deem it inferior to SG [57]. In addition, SG also provides 20-136-fold higher concentration of SN-38 targeted delivery to tumors compared to irinotecan alone [14, 58]. Furthermore, ADCs like SG can also play an immunomodulatory role that can reprogram the tumor immune microenvironment from an immunosuppressive to a pro-inflammatory one, adding to the enhanced efficacy of SG in vivo [59].

It has been previously described that some chemotherapy agents including DNA disruptors and microtubule inhibitors can upregulate the expression of death receptors 4 and 5, increasing cancer cells’ sensitivity to TRAIL [31, 42, 43]. In this study, when surface levels of death receptors were assessed by flow cytometry, there was a two-to-three-fold increase in both DR4 and DR5 expression in SG and combination conditions for MDA-MB-231 and HCC1806 cells. This finding suggests that SG-mediated death receptor upregulation may be a mechanism for its synergy with DR4/DR5 agonist TRAIL. This and other potential mechanisms of synergy including convergence in the downstream effectors of the apoptosis pathway should be explored in future studies.

A number of previous studies have observed significant inhibitory effects with dual treatment with TRAIL and other chemotherapeutic agents including DNA damaging agents in in vitro and in vivo models [31, 42, 43, 60, 61]. Our group has also previously shown that the cell cycle checkpoint regulator WEE1 sensitizes basal breast cancer cells to TRAIL-induced apoptosis [62]. In this current study, we tested possible synergism with DNA damaging agent carboplatin and showed that dual treatment with Apo2L and carboplatin synergistically killed cells in vitro compared to each drug alone. The data suggests that the synergy observed between SG and TRAIL may be applicable to other DNA damaging therapies. Of note, a few clinical trials have shown mixed results for combining TRAIL and DNA damaging agents in humans, with additional studies required including investigations of TRAIL combination with DNA damaging agents in ADC platforms [60, 61].

SG is approved for use in patients with metastatic TNBC who have previously received two or more systemic therapies [11]. Similarly, SG is also approved for use in patients with unrespectable locally advanced or metastatic hormone receptor positive, HER2-negative breast cancer who have received endocrine-based therapy and at least two additional systemic therapies in the metastatic setting [44]. To test whether the SG and Apo2L synergy that’s observed in TNBC can be expanded to the ER+/HER2- subtype, we conducted in vitro viability and cytotoxicity studies. We found that dual treatment with SG and TRAIL synergistically inhibited cell growth in MCF7 cells while synergistically killing cells in T47D ER+/HER2- cell lines. This suggests the potential generalizability of the study’s findings to ER+/HER2- subtype of breast cancer.

While previous work has shown that SG treatment can be effective in Trop2 low in vivo tumor models, this study only examined the effects in vivo in high Trop2 expressing cells [18, 23, 52–54]. Future work utilizing additional xenograft studies with varying levels of Trop2 expression are necessary to further substantiate the efficacy of this combination in vivo. In addition, the tumor pharmacodynamic studies were limited by low and unbalanced sample sizes, which limit the power of analyses and inferences that can be made from the results. In addition, despite being fast growing and sensitive to both SG and Apo2L, the highly ulcerative propensity of the HCC1806 tumor model limited our ability to explore tumor growth and survival for even longer periods of time. Future studies should test combination treatment in additional xenograft models with less propensity for ulcerations. Lastly, ADCs like SG can modulate tumor immune components and TRAIL is also known to affect the tumor microenvironment [59, 63]. Given these observations, evaluating antitumor efficacy of Trop2 directed ADCs and TRAIL agonist combination treatment in immunocompetent models in future studies would provide useful insights including the immune mediated effects. However, we could not test this because SG and the MEDI3039 TRAIL agonist tested in this study do not bind to murine Trop2 and death receptors, respectively [26, 64]. In future studies, the use of humanized murine models (where technically feasible) may be particularly informative for evaluating immune-mediated contributions to the efficacy of combination treatment with SG and TRAIL.

In conclusion, our study suggests that Trop2-directed ADC SG and TRAIL agonists confer a synergistic lethality against TNBC in vitro. Synergistic effect observed in Trop2-low cells is potentially due to extracellular deconjugation of the cytotoxic payload and bystander effects. Dual treatment induces caspases and cell death is rescued by Z-VAD-FMK, indicating that the lethality induced is primarily due to caspase dependent apoptosis. Furthermore, SG alone and in combination with Apo2L induces cell cycle growth arrest in the G2/M phase. Xenograft model data show that combination of SG plus TRAIL agonists induce significant antitumor effects. Overall, our data demonstrate a potential clinical benefit of combination treatment with SG and TRAIL agonists for TNBC.

## Supplementary Information

Below is the link to the electronic supplementary material.

Additional File 1

Additional File 2

Additional File 3

Additional File 4

Additional File 5

Additional File 6

Additional File 7

Additional File 8

Additional File 9

Additional File 10

Additional File 11

Additional File 12

Table S1

Table S2

Table S3

## Data Availability

All data generated or analyzed during this study are included in this published article and its supplementary information files.

## Abbreviations

ADC: Antibody drug conjugate
DR4/DR5: Death receptor 4/5
PDC: Patient derived cells
PDMR: Patient Derived Models Repository
PI: Propidium iodide
SG: Sacituzumab govitecan
TNF: Tumor necrosis factor
TRAIL: Tumor necrosis factor-related apoptosis inducing ligand
Trop2: Trophoblast cell surface antigen 2
Z-VAD-FMK: Carbobenzoxy-valyl-alanyl-aspartyl-[O-methyl]-fluoromethylketone
