# Supplementary material for: Synergistic lethality of combination treatment with Trop2-directed antibody-drug conjugate sacituzumab govitecan and TRAIL agonists in triple negative breast cancer
Source: Breast Cancer Res. 2026 Aug 14;28:144. doi: 10.1186/s13058-026-02318-4 (PMC13474600; doi:10.1186/s13058-026-02318-4)
Supplement: Supplementary file 1 [file 13058_2026_2318_MOESM10_ESM.docx]

**Additional File 10**

***In vitro* synthetic lethality and *in vivo* tolerability of MEDI3039 and SG treatment.** A) HCC1806 cells were treated *in vitro* with SG and TRAIL agonist MEDI3039. Cell death was analyzed using a propidium iodide-based cell death assay 24 hours after treatment. Combination treatment with the two drugs synergistically induced cell death. Data represents the average death (± SEM) of three independent experiments. Synergy scores of repeat experiments were calculated using SynergyFinder. A synergy score of larger than 10 is likely synergistic; from -10 to 10 is likely additive; less than -10 is likely antagonistic. B) In athymic nu/nu nude mice bearing HCC1806 tumors, tolerability of SG and TRAIL agonist MEDI3039 treatment was monitored by body weight measurement twice a week and toxicity limit was set at 15% or more decrease of weight from time of tumor injection. Body weight data is presented as the average (± SD).
