# Supplementary material for: Synergistic lethality of combination treatment with Trop2-directed antibody-drug conjugate sacituzumab govitecan and TRAIL agonists in triple negative breast cancer
Source: Breast Cancer Res. 2026 Aug 14;28:144. doi: 10.1186/s13058-026-02318-4 (PMC13474600; doi:10.1186/s13058-026-02318-4)
Supplement: Supplementary file 2 [file 13058_2026_2318_MOESM12_ESM.docx]

**Additional files**

**Additional file 1**

**Format: .pptx**

**Trop2 expression as measured by immunoblotting in Trop2 low TNBC cell lines.** A higher quantity (40 ug) of protein was run in the Western blot with long exposure of images to evaluate for faint signals as seen in Hs578T. Two blank lanes were used between Hs578T and MDA-MB-231 to prevent the signal from MDA-MB-231 lane obscuring the signal in Hs578T lane during immunoblotting. As indicated by the dashed line, these lanes have been cropped out to fit the figure.

**Additional file 2**

**Format: .pptx**

**Synergistic inhibition of TNBC cells with SG and Apo2L combination treatment.** A-G) TNBC cell lines were treated with Trop2-directed ADC sacituzumab govitecan (SG) and Apo2L (TRAIL agonist). Cell viability was analyzed using an ATP-based cell viability assay for HCC1143, HCC70, BT-20 and Hs578T. Cell death was analyzed using a propidium iodide-based cell death assay with cell imaging performed by BioTek Cytation 1 for SUM-159, MDA-MB-436 and BT-549. Data was collected after 48-hour incubation for all cell lines except HCC1143, SUM-149 and MDA-MB-436, in which data was collected after 24 hours. Data represents the average cell inhibition (± SEM). Combination treatment with the two drugs synergistically inhibited cell viability in all cell lines. Synergy scores of repeat experiments were calculated using SynergyFinder. A synergy score of larger than 10 is likely synergistic; from − 10 to 10 is likely additive; less than − 10 is likely antagonistic.

**Additional file 3**

**Format: .pptx**

**ATP-based cell viability assay for TNBC cells showing synergy between SG and Apo2L.** A-B) Synergy scores of repeat experiments for MDA-MB-468 and MDA-MB-436 were calculated using SynergyFinder. Data represents the average cell inhibition (± SEM). A synergy score of larger than 10 is likely synergistic; from − 10 to 10 is likely additive; less than − 10 is likely antagonistic.

**Additional file 4**

**SN-38 and Apo2L synergistically kill TNBC cells.** A) HCC1806 and MDA-MB-231 cells were treated with SN-38 and Apo2L. Cell death was analyzed either 24 (HCC1806) or 48 (MDA-MB-231) hours later using a propidium iodide-based cell death assay that was read on BioTek Cytation 1. Combination treatment with the two drugs synergistically induced cell death. Data represents the average death (± SEM) of three independent experiments. The top graph shows the data for each cell line and the bottom heat map shows the synergy matrix. C, D) Synergy scores and surface maps of repeat experiments were calculated using SynergyFinder. A synergy score of larger than 10 is likely synergistic; from − 10 to 10 is likely additive; less than − 10 is likely antagonistic.

**Additional file 5**

**Format: .pptx**

**Protocol minimizing SG’s linker deconjugation effect shows synergy with Apo2L in Trop2 high cells only.** A) TNBC cell lines were treated with topoisomerase I inhibitor SN-38 and cell viability was assessed via an ATP-based assay (48 hours). BT-549 cells (low Trop2 expressing) and HCC1806 (medium Trop2 expressing) are cells that have similar sensitivity to SN-38. B) HCC1806 and BT-549 cells were incubated with the Trop2 directed antibody drug conjugate SG for 4 hours after which SG was removed and Apo2L was added. Cell death was assessed via a propidium iodide-based death assay 48 hours later. In this protocol minimizing extracellular deconjugation effects of SN-38 showed synergistic lethality with combination treatment for the Trop2 medium expressing cell line HCC1806 while the Trop2 low expressing BT-549 cells were no longer synergistic. Synergy scores of repeat experiments were calculated using SynergyFinder. A synergy score of larger than 10 is likely synergistic; from − 10 to 10 is likely additive; less than − 10 is likely antagonistic.

**Additional file 6**

**DNA alkylating agent carboplatin synergistically combines with Apo2L to kill TNBC cells through caspase dependent apoptosis.** A, B) TNBC cell lines were treated with SG and Apo2L. Cell death was analyzed using a propidium iodide-based cell death assay with cell imaging performed by BioTek Cytation 1 after 48-hour incubation. C, D) Synergy scores of repeat experiments were calculated using SynergyFinder. A synergy score of larger than 10 is likely synergistic; from − 10 to 10 is likely additive; less than − 10 is likely antagonistic. E, F) Cells were pretreated with cell death inhibitors Z-VAD-FMK, necrostatin and ferrostatin for one hour after which SG and Apo2L drugs were added for 48 hours to assess cell death rescue. Cell death was measured by propidium iodide-based cell death assay. Statistical significance was determined using Student’s *t*-test. * indicates p < 0.05, ** indicates p < 0.01 and *** indicates p < 0.001, **** indicates p < 0.0001. Data is shown as average ± SEM of multiple independent experiments (N ≥ 3).

**Additional file 7**

**Format: .pptx**

**Dose response curves of patient derived TNBC cells treated with SG and Apo2L.** Cell death was analyzed at 24 hours (R015, R081, R296) using a propidium iodide-based cell death assay. Cell viability was assessed after 24 hours using Promega CellTiter-Glo ATP cell viability assay (R207). Data represents the average cell death (± SEM).

**Additional file 8**

**SG and Apo2L dual treatment synergistically inhibits ER+/HER2- cells.** A) Trop2, DR4 and DR5 expression in ER+/HER2- cells MCF-7 and T-47D as measured by immunoblotting. TNBC cell lines MDA-MB-231 and HCC1806 are included for comparison. B, D) Cells were treated with SG and Apo2L. Cell viability was assessed for MCF7 at 24 hours using Promega CellTiter-Glo ATP cell viability assay. C, E) T47D cell death was assessed at 48 hours using a propidium iodide-based cell death assay. Data represents the average death or viability (± SEM) of 3 independent experiments. Synergy scores of repeat experiments were calculated using SynergyFinder. A synergy score of larger than 10 is likely synergistic, from − 10 to 10 is likely additive, less than − 10 is likely antagonistic.

**Additional file 9**

**Format: .pptx**

**Preliminary *in vivo* data of SG and Apo2L combination treatment suggesting positive antitumor effects.** A) Experimental design of *in vivo* study investigating combination treatment with SG and Apo2L. In athymic nu/nu nude mice bearing HCC1806 tumors, SG was administered once a week intraperitonially on day 1 while Apo2L was administered three times a week intraperitonially on days 2–4 at indicated doses. B and C) Tumor sizes were measured twice a week. Two-way ANOVA of tumor growth over time showed significant differences between the combination and control groups. One way ANOVA immediately after two full weeks of treatment showed that average tumor size was significantly decreased with combination treatment with SG and Apo2L, although this anti-tumor effect was lost over time after treatment ended. Longer-term treatment was not investigated due to limitations with Apo2L drug availability. D) Tolerability of SG and Apo2Lcombination treatment as measured by mouse weight. Toxicity limit was set at 15% or more decrease of weight from time of tumor injection. All data represents the average (± SD).

**Additional file 10**

**Format: .pptx**

***In vitro* synthetic lethality and *in vivo* tolerability of MEDI3039 and SG treatment.** A) HCC1806 cells were treated *in vitro* with SG and TRAIL agonist MEDI3039. Cell death was analyzed using a propidium iodide-based cell death assay 24 hours after treatment. Combination treatment with the two drugs synergistically induced cell death. Data represents the average death (± SEM) of three independent experiments. Synergy scores of repeat experiments were calculated using SynergyFinder. A synergy score of larger than 10 is likely synergistic; from − 10 to 10 is likely additive; less than − 10 is likely antagonistic. B) In athymic nu/nu nude mice bearing HCC1806 tumors, tolerability of SG and TRAIL agonist MEDI3039 treatment was monitored by body weight measurement twice a week and toxicity limit was set at 15% or more decrease of weight from time of tumor injection. Body weight data is presented as the average (± SD).

**Additional file 11**

**Format: .pptx**

**Full, uncropped blot images.**

Full uncropped images of the Western blots in the study are included.

The dotted lines in Fig. 1A are added between the low Trop2 expressor and higher Trop2 expressor to prevent the high expressor bleeding into the low lane and obscuring the weaker signal. These lanes were spliced out in the final figures. In Fig. 3B and 3C, the bands for cleaved caspase 3 are marked by the black brackets on both sides of the bands. The bands that appear above are artifacts. In Fig. 5C, the cyclin B1 blot for MDA-MB-468 and Cyclin B1, pHH3 and HSC70 blots for BT-549 are marked with boxes. The bands outside of the indicated boxes are from a separate experiment and, therefore, were spliced out in the final figures. Similarly, in Fig. 6A, the Trop2 and HSC70 bands for the experiment are marked with boxes, the bands outside of the indicated boxes are from a separate experiment and, therefore, were spliced out. For the Trop2 blot, the bands that appear above the Trop2 bands artifacts. On the other hand, the bands for DR5 blot are marked by the black brackets on both sides of the bands; the bands that appear above are artifacts. In Fig. 8, the bands for the respective proteins are marked by brackets, other bands that appear elsewhere are non-specific binding/artifact. In Additional File 1, the dotted lines bracket a blank lane and a MW standard added between low Trop2 expressor and higher Trop2 expressor. These lanes were spliced out in the final figures.
