# Supplementary material for: Synergistic lethality of combination treatment with Trop2-directed antibody-drug conjugate sacituzumab govitecan and TRAIL agonists in triple negative breast cancer
Source: Breast Cancer Res. 2026 Aug 14;28:144. doi: 10.1186/s13058-026-02318-4 (PMC13474600; doi:10.1186/s13058-026-02318-4)
Supplement: Supplementary file 3 [file 13058_2026_2318_MOESM1_ESM.docx]

**Additional File 1**

**Trop2 expression as measured by immunoblotting in Trop2 low TNBC cell lines.** A higher quantity (40 ug) of protein was run in the Western blot with long exposure of images to evaluate for faint signals as seen in Hs578T. Two blank lanes were used between Hs578T and MDA-MB-231 to prevent the signal from MDA-MB-231 lane obscuring the signal in Hs578T lane during immunoblotting. As indicated by the dashed line, these lanes have been cropped out to fit the figure.
