# Supplementary material for: Synergistic lethality of combination treatment with Trop2-directed antibody-drug conjugate sacituzumab govitecan and TRAIL agonists in triple negative breast cancer
Source: Breast Cancer Res. 2026 Aug 14;28:144. doi: 10.1186/s13058-026-02318-4 (PMC13474600; doi:10.1186/s13058-026-02318-4)
Supplement: Supplementary file 4 [file 13058_2026_2318_MOESM2_ESM.docx]

**Additional File 2**

**Synergistic inhibition of TNBC cells with SG and Apo2L combination treatment.** A-G) TNBC cell lines were treated with Trop2-directed ADC sacituzumab govitecan (SG) and Apo2L (TRAIL agonist). Cell viability was analyzed using an ATP-based cell viability assay for HCC1143, HCC70, BT-20 and Hs578T. Cell death was analyzed using a propidium iodide-based cell death assay with cell imaging performed by BioTek Cytation 1 for SUM-159, MDA-MB-436 and BT-549. Data was collected after 48-hour incubation for all cell lines except HCC1143, SUM-149 and MDA-MB-436, in which data was collected after 24 hours. Data represents the average cell inhibition (± SEM). Combination treatment with the two drugs synergistically inhibited cell viability in all cell lines. Synergy scores of repeat experiments were calculated using SynergyFinder. A synergy score of larger than 10 is likely synergistic; from -10 to 10 is likely additive; less than -10 is likely antagonistic.
