# Supplementary material for: Synergistic lethality of combination treatment with Trop2-directed antibody-drug conjugate sacituzumab govitecan and TRAIL agonists in triple negative breast cancer
Source: Breast Cancer Res. 2026 Aug 14;28:144. doi: 10.1186/s13058-026-02318-4 (PMC13474600; doi:10.1186/s13058-026-02318-4)
Supplement: Supplementary file 5 [file 13058_2026_2318_MOESM3_ESM.docx]

**Additional File 3**

**ATP-based cell viability assay for TNBC cells showing synergy between SG and Apo2L.** A-B) Synergy scores of repeat experiments for MDA-MB-468 and MDA-MB-436 were calculated using SynergyFinder. Data represents the average cell inhibition (± SEM). A synergy score of larger than 10 is likely synergistic; from -10 to 10 is likely additive; less than -10 is likely antagonistic.
