# Supplementary material for: Synergistic lethality of combination treatment with Trop2-directed antibody-drug conjugate sacituzumab govitecan and TRAIL agonists in triple negative breast cancer
Source: Breast Cancer Res. 2026 Aug 14;28:144. doi: 10.1186/s13058-026-02318-4 (PMC13474600; doi:10.1186/s13058-026-02318-4)
Supplement: Supplementary file 6 [file 13058_2026_2318_MOESM4_ESM.docx]

**Additional File 4**

**SN-38 and Apo2L synergistically kill TNBC cells.** A) HCC1806 and MDA-MB-231 cells were treated with SN-38 and Apo2L. Cell death was analyzed either 24 (HCC1806) or 48 (MDA-MB-231) hours later using a propidium iodide-based cell death assay that was read on BioTek Cytation 1. Combination treatment with the two drugs synergistically induced cell death. Data represents the average death (± SEM) of three independent experiments. The top graph shows the data for each cell line and the bottom heat map shows the synergy matrix. C, D) Synergy scores and surface maps of repeat experiments were calculated using SynergyFinder. A synergy score of larger than 10 is likely synergistic; from -10 to 10 is likely additive; less than -10 is likely antagonistic.
