# Supplementary material for: Synergistic lethality of combination treatment with Trop2-directed antibody-drug conjugate sacituzumab govitecan and TRAIL agonists in triple negative breast cancer
Source: Breast Cancer Res. 2026 Aug 14;28:144. doi: 10.1186/s13058-026-02318-4 (PMC13474600; doi:10.1186/s13058-026-02318-4)
Supplement: Supplementary file 7 [file 13058_2026_2318_MOESM5_ESM.docx]

**Additional File 5**

**Protocol minimizing SG’s linker deconjugation effect shows synergy with Apo2L in Trop2 high cells only.** A) TNBC cell lines were treated with topoisomerase I inhibitor SN-38 and cell viability was assessed via an ATP-based assay (48 hours). BT-549 cells (low Trop2 expressing) and HCC1806 (medium Trop2 expressing) are cells that have similar sensitivity to SN-38. B) HCC1806 and BT-549 cells were incubated with the Trop2 directed antibody drug conjugate SG for 4 hours after which SG was removed and Apo2L was added. Cell death was assessed via a propidium iodide-based death assay 48 hours later. This protocol minimizing extracellular deconjugation effects of SN-38 showed synergistic lethality with combination treatment for the Trop2 medium expressing cell line HCC1806 while the Trop2 low expressing BT-549 cells were no longer synergistic. Synergy scores of repeat experiments were calculated using SynergyFinder. A synergy score of larger than 10 is likely synergistic; from -10 to 10 is likely additive; less than -10 is likely antagonistic.
