# Supplementary material for: Synergistic lethality of combination treatment with Trop2-directed antibody-drug conjugate sacituzumab govitecan and TRAIL agonists in triple negative breast cancer
Source: Breast Cancer Res. 2026 Aug 14;28:144. doi: 10.1186/s13058-026-02318-4 (PMC13474600; doi:10.1186/s13058-026-02318-4)
Supplement: Supplementary file 8 [file 13058_2026_2318_MOESM6_ESM.docx]

**Additional File 6**

**DNA alkylating agent carboplatin synergistically combines with Apo2L to kill TNBC cells through caspase dependent apoptosis.** A, B) TNBC cell lines were treated with carboplatin and Apo2L. Cell death was analyzed using a propidium iodide-based cell death assay with cell imaging performed by BioTek Cytation 1 after 48-hour incubation. C, D) Synergy scores of repeat experiments were calculated using SynergyFinder. A synergy score of larger than 10 is likely synergistic; from -10 to 10 is likely additive; less than -10 is likely antagonistic. E, F) Cells were pretreated with cell death inhibitors Z-VAD-FMK, necrostatin and ferrostatin for one hour after which SG and Apo2L drugs were added for 48 hours to assess cell death rescue. Cell death was measured by propidium iodide-based cell death assay. Statistical significance was determined using Student’s *t*-test. * indicates p<0.05, ** indicates p<0.01 and *** indicates p<0.001, **** indicates p<0.0001. Data is shown as average ± SEM of multiple independent experiments (N ≥ 3).
