# Supplementary material for: Synergistic lethality of combination treatment with Trop2-directed antibody-drug conjugate sacituzumab govitecan and TRAIL agonists in triple negative breast cancer
Source: Breast Cancer Res. 2026 Aug 14;28:144. doi: 10.1186/s13058-026-02318-4 (PMC13474600; doi:10.1186/s13058-026-02318-4)
Supplement: Supplementary file 9 [file 13058_2026_2318_MOESM7_ESM.docx]

**Additional File 7**

**7**

**Dose response curves of patient derived TNBC cells treated with SG and Apo2L.** Cell death was analyzed at 24 hours (R015, R081, R296) using a propidium iodide-based cell death assay. Cell viability was assessed after 24 hours using Promega CellTiter-Glo ATP cell viability assay (R207). Data represents the average cell death (± SEM).
