# Supplementary material for: Synergistic lethality of combination treatment with Trop2-directed antibody-drug conjugate sacituzumab govitecan and TRAIL agonists in triple negative breast cancer
Source: Breast Cancer Res. 2026 Aug 14;28:144. doi: 10.1186/s13058-026-02318-4 (PMC13474600; doi:10.1186/s13058-026-02318-4)
Supplement: Supplementary file 10 [file 13058_2026_2318_MOESM8_ESM.docx]

**Additional File 8**

**SG and Apo2L dual treatment synergistically inhibits ER+/HER2- cells.** A) Trop2, DR4 and DR5 expression in ER+/HER2- cells MCF7 and T47D as measured by immunoblotting. TNBC cell lines MDA-MB-231 and HCC1806 are included for comparison. B, D) Cells were treated with SG and Apo2L. Cell viability was assessed for MCF7 at 24 hours using Promega CellTiter-Glo ATP cell viability assay. C, E) T47D cell death was assessed at 48 hours using a propidium iodide-based cell death assay. Data represents the average death or viability (± SEM) of 3 independent experiments. Synergy scores of repeat experiments were calculated using SynergyFinder. A synergy score of larger than 10 is likely synergistic, from -10 to 10 is likely additive, less than -10 is likely antagonistic.
