# Supplementary material for: Synergistic lethality of combination treatment with Trop2-directed antibody-drug conjugate sacituzumab govitecan and TRAIL agonists in triple negative breast cancer
Source: Breast Cancer Res. 2026 Aug 14;28:144. doi: 10.1186/s13058-026-02318-4 (PMC13474600; doi:10.1186/s13058-026-02318-4)
Supplement: Supplementary file 11 [file 13058_2026_2318_MOESM9_ESM.docx]

**Additional File 9**

**Preliminary *in vivo* data of SG and Apo2L combination treatment suggesting positive antitumor effects.** A) Experimental design of *in vivo* study investigating combination treatment with SG and Apo2L. In athymic nu/nu nude mice bearing HCC1806 tumors, SG was administered once a week intraperitonially on day 1 while Apo2L was administered three times a week intraperitonially on days 2-4 at indicated doses. B and C) Tumor sizes were measured twice a week. Two-way ANOVA of tumor growth over time showed significant differences between the combination and control groups. One way ANOVA immediately after two full weeks of treatment showed that average tumor size was significantly decreased with combination treatment with SG and Apo2L, although this anti-tumor effect was lost over time after treatment ended. Longer-term treatment was not investigated due to limitations with Apo2L drug availability. D) Tolerability of SG and Apo2L combination treatment as measured by mouse weight. Toxicity limit was set at 15% or more decrease of weight from time of tumor injection. All data represents the average (± SD).
